# Removal of contaminants by activating peroxymonosulfate (PMS) using zero valent iron (ZVI)-based bimetallic particles (ZVI/Cu, ZVI/Co, ZVI/Ni, and ZVI/Ag)†

**DOI:** 10.1039/d0ra03924a

**Published:** 2020-07-28

**Authors:** Xiaowei Huo, Peng Zhou, Yunxin Liu, Feng Cheng, Yang Liu, Xin Cheng, Yongli Zhang, Qingguo Wang

**Affiliations:** College of Architecture & Environment, Sichuan University Chengdu 610065 PR China xyl_scu@126.com wangqgscu@163.com; Department of Chemical and Environmental Engineering, Yale University New Haven Connecticut 06511 USA

## Abstract

In this study, four different ZVI/M-PMS systems (*e.g.*, ZVI/Cu, ZVI/Co, ZVI/Ni and ZVI/Ag) were fabricated to investigate the removal of contaminants (Rhodamine B (RB), 2,4-dichlorophenol (2,4-DCP), bisphenol A (BPA), bisphenol F (BPF), levofloxacin (LFX), and chloramphenicol (CAP)). The results indicated that ZVI/Cu and ZVI/Ag exhibited a superior performance to activate PMS compared with the ZVI. The mechanism of the investigation showed that a relatively positive correlation between the release of iron ions (Fe^2+^) and contaminant removal efficiency was found in different ZVI/M-PMS systems. This revealed that galvanic couples affected iron corrosion, and the ZVI/Cu and ZVI/Ag bimetallic systems facilitated the corrosion of ZVI but the ZVI/Co and ZVI/Ni bimetallic systems restrained the corrosion of ZVI. The electron paramagnetic resonance (EPR) analysis and the radical quenching experiments apparently supported the roles of the hydroxyl radical (˙OH), sulfate radical (SO_4_˙^−^) and superoxide radicals (O_2_˙^−^), which suggest that these reactive radicals were primarily responsible for the degradative route, and the contribution rate may follow the order of SO_4_˙^−^ < O_2_˙^−^ < ˙OH. Furthermore, investigation of crucial parameters showed that the contaminant removal ratio increased with an increase in the metal ratio (M : ZVI) to a certain limit, and a higher bimetal catalyst dosage and extremely acidic conditions (except for ZVI/Co, which showed the best catalytic performance under neutral condition) enhanced the degradation of contaminants. In the evaluation of real water samples, there was almost no influence from the water matrices compared to the control condition, and the ZVI/Cu and ZVI/Ag bimetallic particles showed great potential to treat various wastewater. Therefore, this study helps to understand the application of oxidation process based on bimetallic particles.

## Abstract 2

In this study, four different ZVI/M-PMS systems (*e.g.*, ZVI/Cu, ZVI/Co, ZVI/Ni and ZVI/Ag) were fabricated to investigate the removal of contaminants (Rhodamine B, 2,4-dichlorophenol, bisphenol A, bisphenol F, levofloxacin, and chloramphenicol).

## Graphical abstract

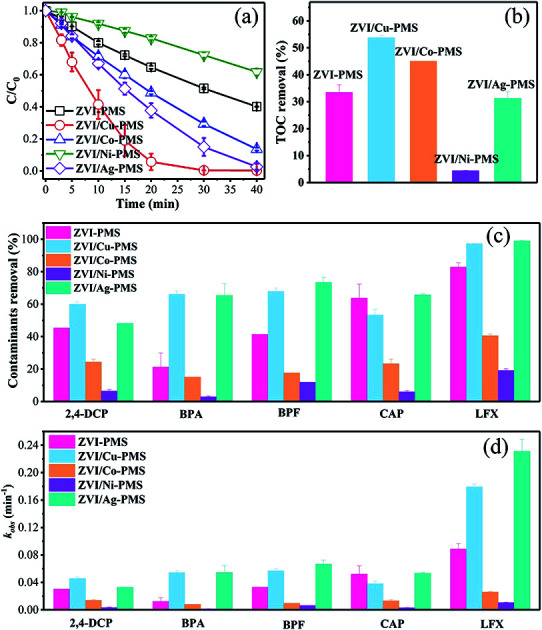

## 1. Introduction

Nowadays, with the increasing demand for synthetic organic chemicals such as RB, which is a distinct representative of organic dyes and a laser material in the paper-making, textile, and photographic industries having high stability;^1^ 2,4-DCP, which is a chlorinated derivative of phenol used in wide variety of applications including dye intermediate and medical disinfectant;^2^ BPA, which is used in plastics, receipts, food packaging, and other products that might be harmful to human health due to its actions as an endocrine-disrupting chemical;^3^ BPF, which is used in the manufacture of epoxy resins and coatings, and in polymers that give materials increased thickness and durability;^4^ LFX, which is a fluoroquinolone-type antibiotic widely employed for the treatment of various diseases associated with bacterial infections with 44 million prescriptions annually;^5^ and CAP, which is a broad-spectrum antibiotic and has a good inhibitory effect on Brucella, Gram-positive bacteria, Gram-negative bacteria, Rickettsia and Chlamydia,^6^ the requirements for water treatment technologies have also increased.

As an efficient method for degrading various organic pollutants, advanced oxidation processes (AOPs) have attracted extensive attention owing to their ability to generate reactive radicals such as ˙OH and SO_4_˙^−^.^7^ The reactive radicals exhibit advantages such as high redox potential, long lifetime and stable structures.^8,9^ PMS (HSO_5_^−^) and peroxydisulfate (PDS, S_2_O_8_^2−^) are usually used as precursors to produce reactive radicals *via* the ultraviolet light,^10^ ultrasound,^11^ heat,^12^ base,^13^ transition metals,^14^ and carbon-based materials.^15^

Previous studies have demonstrated that ZVI as a representative transition metal exhibits good activation performance for PMS. Jinyao Cao *et al.* investigated the removal of tetracycline with the ZVI-PMS system.^8^ Also, Jiayi Yao *et al.* reported the performance of ZVI-activated PMS for the removal of bisphenol M.^16^ Moreover, ZVI possesses good properties and is inexpensive, environment-friendly and easily available.^17^ However, ZVI suffers from the limitations of a low reaction rate and low removal rate on account of its surface passivation and narrow reactive pH range.^18^ Accordingly, the reactivity of ZVI can be enhanced *via* pretreatment (acid washing, ultrasound, and pre-magnetization), physical technology (UV-visible light and microwaves), chemical technology (doped with Co^2+^ and Mn^2+^), *etc.*^19,20^

Bimetallic particles are widely considered promising materials for the degradation of organic contaminants.^21–23^ Yang Sun *et al.* reported that Fe/Cu bimetallic catalysts enhanced the generation of ˙OH and degradation of efficiency of nitrobenzene.^24^ Yu Wang *et al.* also explored the influence of Cu(ii), Ni(ii), and Zn(ii) ions in the nZVI-PS system on the degradation of organic contaminants. They found that Cu(ii) enhanced the degradation of 2,2′,4,4′-tetrabromodiphenyl ether (BDE-47), which is an extensively used brominated flame retardant with carcinogen properties; however, Ni(ii) and Zn(ii) inhibited the degradation of BDE-47, which may because of the negative reduction potentials of Ni(ii) and Zn(ii), leading to surface adsorption on or complexation with nZVI.^25^ Rui Wang *et al.* demonstrated that in the Fe/Ag system, electron transfer is the dominant mechanism, whereas H atom transfer is the dominant mechanism in the Fe/Pd system.^26^ Considering that some ZVI-based bimetal particles have better catalytic performances (greater removal ability, higher reaction rate and wider reaction pH range) than ZVI, it is reasonable to speculate that ZVI-based bimetals will be more effective and faster to active PMS for the degradation of pollutants.

To investigate the efficiency of removing organic compounds with bimetal/PMS systems, we explored bimetal/PMS systems as follows: (i) the properties of four different bimetallic particles (ZVI/Cu, ZVI/Co, ZVI/Ni, and ZVI/Ag) were characterized *via* SEM, EDS, XPS and XRD; (ii) the mechanism of the bimetal/PMS system was studied *via* EPR analysis, quenching tests and metal transformation; and (iii) the effect of crucial parameters including metal ratio, dosage of bimetallic particles, initial pH and water matrix was determined.

## 2. Materials and methods

### 2.1 Materials

The materials were of analytical reagent grade, which are shown in the ESI (Text S1).†

### 2.2 Preparation of the bimetallic particles

The bimetallic particles (ZVI/Cu, ZVI/Co, ZVI/Ni, and ZVI/Ag) were prepared based on a replacement reaction. Firstly, the required ZVI powder was added to 250 mL of metal solution (CuSO_4_, CoSO_4_, NiSO_4_, and AgNO_3_), and mixed for 15 min with mechanical stirrer at a speed of 250 rpm. Secondly, the obtained products were separated after 3 min precipitation. Finally, the isolated products were washed several times with ultra-pure water, and then dried in a freezing-vacuum dryer for 8 h.^27^ The previous studies revealed that the inert metal mass loading on the surface of ZVI influences the catalytic activity of the prepared bimetallic particles.^28^ Also, the inert metal (Cu, Co, Ni, and Ag) mass loading of the bimetallic particles could be changed by adjusting the concentration of inert metal solution. The solution concentration according to the molar ratio (M : ZVI) was 1 : 5, 1 : 10 and 1 : 50.

### 2.3 Experimental procedure

All the experiments were carried out in 500 mL ultra-pure water under constant mechanical stirring (500 rpm). The initial pH value was adjusted with 1 M H_2_SO_4_ and 1 M NaOH. Each run was started by adding the desired dosage of contaminants, PMS and bimetallic particles simultaneously. The concentrations of contaminants were detected at pre-scheduled intervals after filtration with a 0.22 μm PTFE membrane. The residual bimetallic particles were withdrawn, and vacuum filtered, then dried in a freezing-vacuum dryer for further characterization.

### 2.4 Analytical procedures

The concentration of Rhodamine B (RB) was determined using a UV-1800 spectrophotometer at 544 nm. The concentration of 2,4-DCP, BPA, BPF, LFX and CAP in aqueous solution was analyzed by HPLC chromatography (Text S2).† The pH value was monitored using a pH meter (FE-28 standard). The total organic carbon (TOC) of the sample was analyzed using a multi N/C 3100 analyzer (Analytik Jena). Atomic absorption spectrometry (ICPOES730, Agilent) was employed to determine the dissolved metal ions. More detailed information about the characterization is presented Text S3.† EPR experiments were performed to analyze ˙OH, SO_4_˙^−^ and O_2_˙^−^ with DMPO as the spin-trapping agent (Text S4).† The open-circuit potential curves were measured on an electrochemical workstation (CHI 660E, China). The photometric detection of Fe(iv) is described in Text S5.†

## 3. Results and discussion

### 3.1 Removal of various organics in bimetallic particle-mediated PMS activation

#### 3.1.1 Discussion of RB degradation

**Fig. 1:**
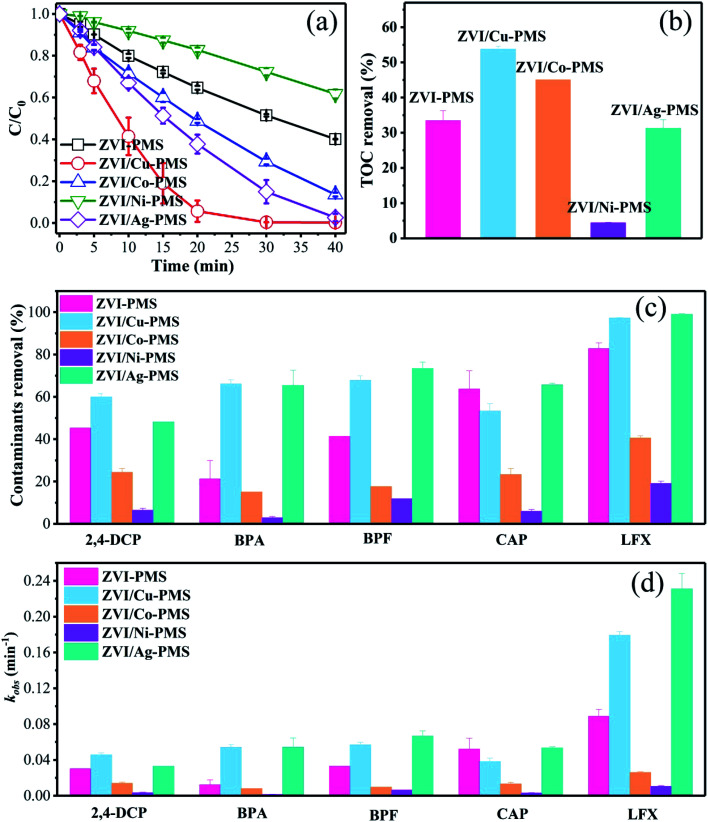
(a) RB degradation in the different systems. (b) TOC (RB, reaction time 3 h) degradation in the different systems. [RB]_0_ = 20 mg L^−1^, [PMS]_0_ = 1 mM, [PMS] : [RB] = 15.4 : 1, [catalyst]_0_ = 100 mg L^−1^, initial pH = 3 ± 0.2, *T* = 25 ± 1 °C. (c) Degradation of various organic compounds in the different systems within 20 min. (d) Reaction rates for the different systems within 20 min. [2,4-DCP]_0_ = [BPA]_0_ = [BPF]_0_ = [CAP]_0_ = [LFX]_0_ = 20 mg L^−1^, [PMS]_0_ = 1 mM, [PMS] : [organic contaminant] = 15.4 : 1, [catalyst]_0_ = 100 mg L^−1^, initial pH = 3 ± 0.2, *T* = 25 ± 1 °C.

**Fig. 2:**
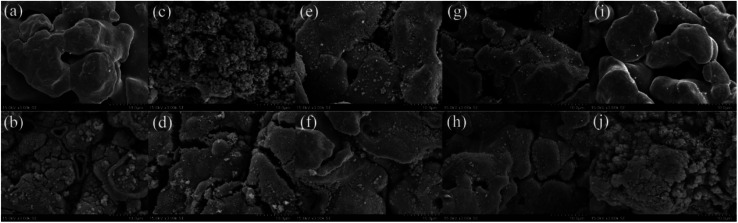
SEM micrographs before and after the reaction of (a and b) ZVI-PMS system, (c and d) ZVI/Cu-PMS system, (e and f) ZVI/Co-PMS system, (g and h) ZVI/Ni-PMS system and (i and j) ZVI/Ag-PMS system, respectively. [RB]_0_ = 20 mg L^−1^, [PMS]_0_ = 1 mM, [PMS] : [RB] = 15.4 : 1, [catalyst]_0_ = 100 mg L^−1^, initial pH = 3 ± 0.2, *T* = 25 ± 1 °C.

To preliminarily probe the catalytic reactivity of ZVI/M for activating PMS (M represents Cu, Co, Ni and Ag), the degradation of various organic pollutants was demonstrated. As shown in Fig. 1(a), the ZVI/Cu-PMS system exhibited the highest RB removal ratio of 99.78% within 40 min and the fastest apparent rate constant (*k*_obs_) of 0.195 min^−1^ (Fig. S1†). In the ZVI/Co-PMS system and the ZVI/Ag-PMS system, the removal ratio of RB was 86.42% and 97.43%, respectively. The above systems exhibited a higher RB removal ratio than the ZVI-PMS system of 59.87%. In contrast, RB was degraded slightly with the removal ratio of only 38.25% in the ZVI/Ni-PMS system, which showed an inhibitory effect on removing RB compared with the ZVI-PMS system. As shown in Fig. 2, the TOC removal at 3 h reached the maximum in the ZVI/Cu-PMS system of 53.8%, which exactly coincided with the highest RB removal and the fastest reaction rate. In the ZVI-PMS, ZVI/Co-PMS and ZVI/Ag-PMS systems, the removal ratios of TOC were 33.4%, 45.0% and 31.3%, respectively. The lowest TOC removal of 4.4% was observed in the ZVI/Ni-PMS system.

#### 3.1.2 Degradation of other organic compounds

As is known, different chemical compositions and structures may affect the efficiency of catalytic systems. Zhou *et al.* found that the removal efficiencies of acid orange 7 and RB were clearly lower in nano/micron zero valent copper-PMS systems compared to other treatments, perhaps because of the direct Sandmeyer reaction between Cu^+^ and the dyes, which reduced the availability of Cu^+^ to activate PMS.^29,30^ However, in our ZVI/Cu-PMS system and ZVI/Ag-PMS system, the degradation efficiency of various organic contaminants was the same. As shown in Fig. 1(c), (d), S2 and Table S1,† five organic compounds with different molecular structures were tested under the same conditions, including 2,4-DCP, BPA, BPF, CAP and LFX. Fig. S2† shows that the ZVI/Cu-PMS system and the ZVI/Ag-PMS system exhibited a good degradation efficiency for these five organic compounds, which were almost completely degraded after 40 min. It is worth noting that the *k*_obs_ for BPA and LFX in the ZVI/Cu-PMS system was 0.054 and 0.179 min^−1^, and in the ZVI/Ag-PMS system 0.054 and 0.231 min^−1^ within 20 min, compared with 0.0122 and 0.089 min^−1^ for the ZVI-PMS system, respectively. In general, when PMS was added, the ZVI-PMS system exhibited selective removal capacity towards 2,4-DCP, CAP, and LFX at 40 min, but the ZVI/Cu-PMS and ZVI/Ag-PMS systems exhibited effective degradation efficiency for the five organic compounds. Nevertheless, the removal efficiencies of the five organic compounds were clearly lower in the ZVI/Co-PMS and ZVI/Ni-PMS systems.

Comparing six types of organic compounds, it is worth noting that the RB degradation efficiency in the five systems tended to differentiate more easily than the other five organic compounds. Hence, this study investigated RB degradation efficiency with the five systems. All the results revealed that the RB degradation rate was not only enhanced, but the TOC removal efficiency was also improved by using the bimetallic particles (ZVI/Cu, ZVI/Co, and ZVI/Ag) rather than ZVI to activate PMS.

### 3.2 Mechanism discussion

#### 3.2.1 Characterization of bimetallic powders

As shown in Fig. 2, the SEM micrographs revealed that the pristine ZVI had a very smooth surface, while the residual ZVI became obviously rough and porous after the reaction. The prepared ZVI/Cu catalyst had a fluffy uneven appearance, which may be attributed to the high intensity of the replacement reaction of ZVI and Cu^2+^, and after the reaction, its surface became tight and porous. It can be observed in Fig. 1(a) that the ZVI/Cu-PMS system exhibited the best performance among the systems, which may be due to the Cu layer assisting the electron transfer.^31^ Furthermore, the accumulation of Cu^+^ on the ZVI/Cu surface may provide abundant reactive species.^32^ The prepared ZVI/Co, ZVI/Ni and ZVI/Ag catalysts appeared to have some granular matters stuck on their surface, and the reacted catalysts showed a corroded surface to different degrees. The facial roughness of the residual catalysts was related to their catalytic performance, where the changes were greater before and after reaction for the catalysts with stronger activation ability.

**Fig. 3:**
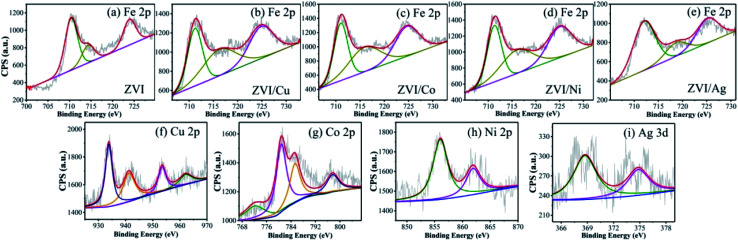
XPS spectra of (a–e) Fe, (f) Cu, (g) Co, (h) Ni and (i) Ag in the different bimetallic particles.

The elemental composition and oxidation states of the bimetallic particles were further elucidated *via* XPS. Fig. S8† shows the XPS full-scan spectra, which reveal the main elements present in the surface of the five catalysts, including O, Fe and the specific metal (Cu/Co/Ni/Ag). The Fe 2p spectra (Fig. 3(a)) show peaks at 711.2 eV, 714.5 eV and Fe 724.8 eV, which correspond to the Fe(iii) Fe 2p_3/2_, satellite of Fe(ii) Fe 2p_3/2_ and Fe(ii) Fe 2p_1/2_, respectively,^33,34^ indicating that the outer surface of ZVI was covered by a layer of oxide film. Compared with Fig. 3(a), Fig. 3(b)–(e) show the peaks of Fe 2p present in the different bimetallic particles, which reveal that the bimetallic particles could affect the formation of Fe(ii).^34^ As shown in Fig. 3(f), the peaks at 933.9 eV and 953.6 eV are attributed to Cu 2p_3/2_ and Cu 2p_1/2_ of Cu^0^, respectively, and the other two peaks located at 941.8 eV and 962.3 eV are associated with Cu^2+^, which evidence the presence of CuO.^35^ In the Co 2p spectra (Fig. 3(g)), the two sharp peaks at 780.8 eV and 797.6 eV with the satellite peaks at 785.3 eV and 772.2 eV can be assigned to Co 2p_3/2_ and Co 2p_1/2_, respectively, which correspond to Co(ii).^36^Fig. 3(h) displays two peaks including Ni 2p_3/2_ and Ni 2p_1/2_ at 855.9 eV and 561.3 eV, respectively. Thus, Ni was in the form of +2 and +3 valent states.^37^ As shown in Fig. 3(i), the Ag 3d_5/2_ (368.5 eV) and Ag 3d_3/2_ (374.7 eV) peaks was associated with Ag^0^, indicating that only metallic Ag existed on the surface of the bimetallic particles.^38^

**Fig. 4:**
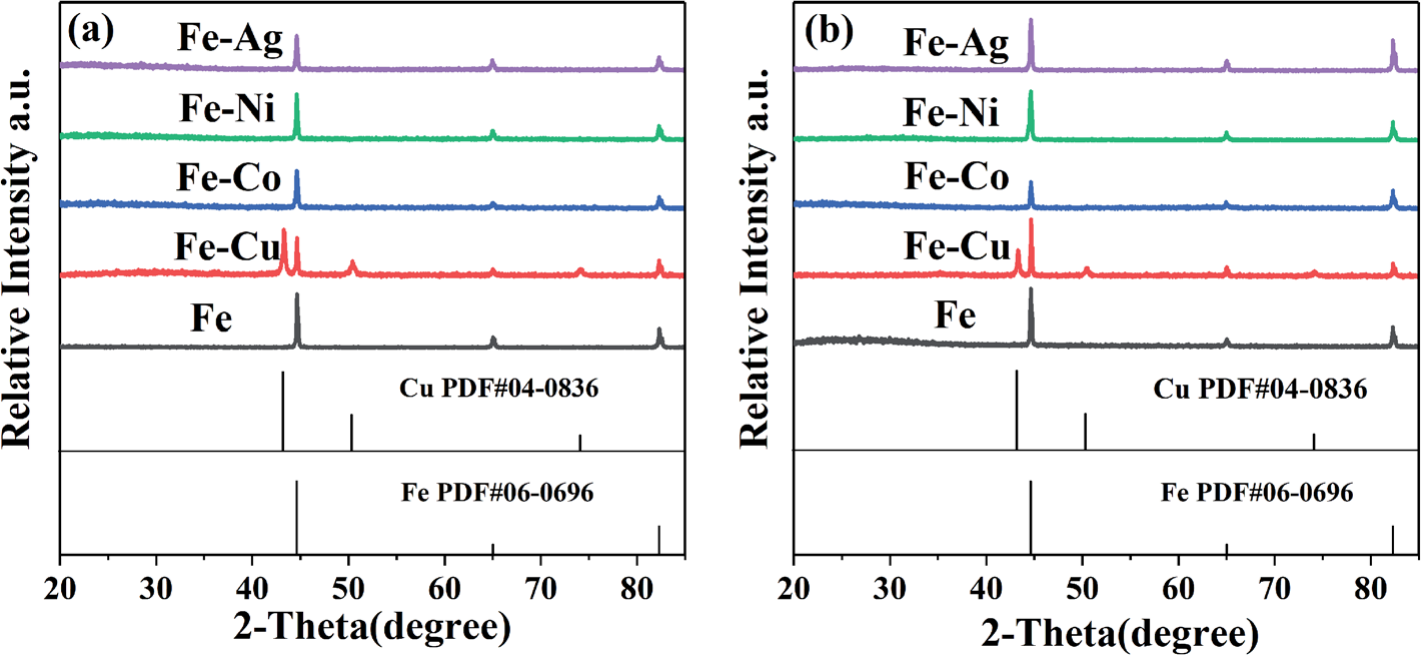
XRD patterns of the bimetallic particles (a) before reaction and (b) after reaction. [RB]_0_ = 20 mg L^−1^, [PMS]_0_ = 1 mM, [PMS] : [RB] = 15.4 : 1, [catalyst]_0_ = 100 mg L^−1^, initial pH = 3 ± 0.2, *T* = 25 ± 1 °C.

The ZVI and bimetallic particles before and after the reaction were analyzed *via* XRD to confirm the generated substances during the preparation process and reaction. Only two substances can be identified in Fig. 4, which are Fe^0^ and Cu^0^. Thus, the XRD patterns are in good agreement with the standard data of ZVI (PDF #06-0696) and Cu^0^ (PDF #04-0836). No Co, Ni, and Ag were found in the XRD spectra, which may be related to their low mass loading on the surface of these inert metals, according to Table 1.

**Table 1:** The inert metal mass loading (weight%) on the surface of the catalysts with different molar ratios

M : ZVI	ZVI/Cu	ZVI/Co	ZVI/Ni	ZVI/Ag
1 : 5	76.17	2.01	2.23	Not detected
1 : 10	73.26	1.91	1.21	Not detected
1 : 50	29.83	0.12	0.47	Not detected

#### 3.2.2 Generation of reactive oxidants

**Fig. 5:**
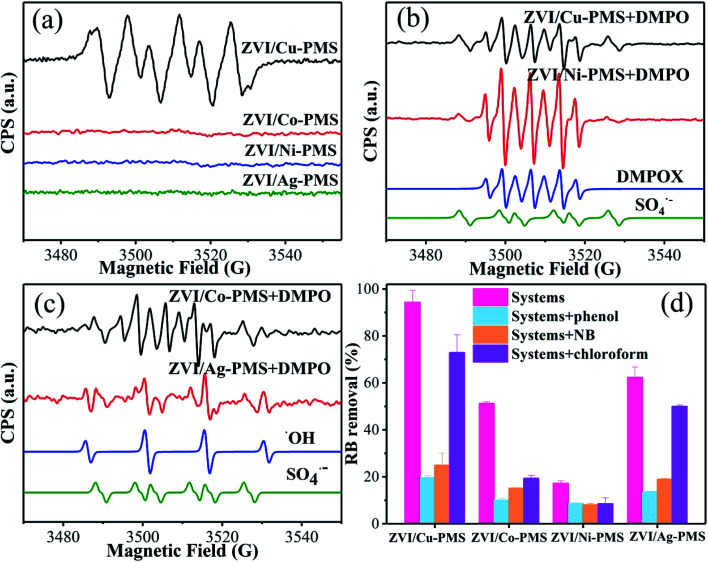
(a) EPR spectra analysis in methanol solvent. (b and c) EPR spectra analysis in aqueous solvent. [PMS]_0_ = 1 mM, [catalyst]_0_ = 100 mg L^−1^, [DMPO] = 10 mM. (d) RB removal in the different reaction systems with or without inhibitors within 20 min. [RB]_0_ = 20 mg L^−1^, [PMS]_0_ = 1 mM, [PMS] : [RB] = 15.4 : 1, [catalyst]_0_ = 100 mg L^−1^, [phenol]_0_ = [NB]_0_ = [Chloroform]_0_ = 10 mM, initial pH = 3 ± 0.2, *T* = 25 ± 1 °C.

Previous studies mentioned that PMS activation by metals may generate reactive radicals,^29^ and it is well known that ˙OH, SO_4_˙^−^ and O_2_˙^−^ can be indirectly detected by EPR spectroscopy with the spin-trapping reagent DMPO. Also, the qualitative analysis of ˙OH, SO_4_˙^−^ and HO_2_˙ can be achieved due to the signals of DMPO–SO_4_,^39^ DMPO–OH^1,40^ and DMPO–HO_2_.^40,41^Fig. 5(a)–(c) show the EPR spectra for the different systems after the addition of PMS. It is known that HO_2_˙ is the main oxidative species formed under pH 3.2; however, Fig. 5(a) shows that the six characteristic peaks of DMPO–HO_2_ were only obtained in methanol solvent for the ZVI/Cu-PMS system, confirming the formation of O_2_˙^−^ during the reaction.^42^ Based on the mechanism of O_2_˙^−^ formation in previous studies,^32,42,43^ we speculated that O_2_˙^−^ generation in the ZVI/M-PMS system (M represents Cu, Co, Ni and Ag) follows eqn (1)–(7). As shown in Fig. 5(b) and (c), in the ZVI/Cu-PMS, ZVI/Co-PMS and ZVI/Ag-PMS systems, SO_4_˙^−^ was produced due to the signals of the DMPO–SO_4_ adducts, indicating that PMS was activated by ZVI/Cu, ZVI/Co and ZVI/Ag to generate SO_4_˙^−^ immediately. Fig. 5(b) and (c) also indicate that the characteristic heptet peaks were detected in the spectrum. These results reveal that DMPO was oxidized to DMPOX by oxidizing species.^44^ For comparison, in the ZVI/Ag-PMS system, SO_4_˙^−^ and ˙OH were generated with 2 min, demonstrating that ˙OH can be produced by HSO_5_^−^ and SO_4_˙^−^*via*eqn (8)–(12) in the systems.^8,45,46^

1 Fe^0^ + 2Cu^2+^ + 2H_2_O → Fe^2+^ + Cu_2_O + H^+^

2 2Cu^0^ + HSO_5_^−^ → 2Cu^+^ + SO_4_^2−^ + OH^−^

3 HSO_5_^−^ + H_2_O → HSO_4_^−^ + H_2_O_2_

4 Fe^2+^ + O_2_ → O_2_˙^−^ + Fe^3+^

5 Fe^3+^ + H_2_O_2_ → HO_2_˙ + Fe^2+^ + H^+^

6 HO_2_˙ → O_2_˙^−^ + H^+^

7 Cu^+^ + O_2_ → O_2_˙^−^ + Cu^2+^

8 HSO_5_^−^ + Fe^2+^ → SO_4_˙^−^ + Fe^3+^ + OH^−^

9 HSO_5_^−^ + Fe^2+^ → ˙OH + Fe^3+^ + SO_4_^2−^

10 SO_4_˙^−^ + OH^−^ → ˙OH + SO_4_^2−^

11 H_2_O + SO_4_˙^−^ → SO_4_^2−^ + ˙OH + H^+^

12 2H_2_O + HSO_5_^−^ → SO_4_^2−^ + 2˙OH + H^+^

Moreover, phenol is a forceful quencher for both ˙OH and SO_4_˙^−^ (*k*(Phenol/˙OH) = 6.6 × 10^9^ M^−1^ s^−1^, *k*(Phenol/SO_4_˙^−^) = 8.8 × 10^9^ M^−1^ s^−1^), while NB can only quench ˙OH in aqueous solution (*k*(NB/˙OH) = 3.0–3.9 × 10^9^ M^−1^ s^−1^, *k*(NB/SO_4_˙^−^) < 10^6^ M^−1^ s^−1^).^47^ Also, trichloromethane is a powerful inhibitor for O_2_˙^−^ (*k*(CHCl_3_/O_2_˙^−^) < 10^6^ M^−1^ s^−1^).^48^ As shown in Fig. 5(d), the removal of RB was obviously reduced in all the treatment systems (especially in the ZVI/Cu system) when phenol, NB, or trichloromethane were added. Meanwhile, there was some additional difference in the RB removal efficiency between adding phenol and NB. As shown in Fig. 5(d), the RB degradation was 94.3% for the ZVI/Cu-PMS system, 51.2% for the ZVI/Co-PMS system, 17.2% for the ZVI/Ni-PMS system and 62.3% for the ZVI/Ag-PMS system. However, it decreased to 19.6%, 10.0%, 8.7% and 13.6% in the presence of 10 mM phenol and 24.9%, 15.1%, 8.1% and 18.9% in the presence of 10 mM NB, respectively, which demonstrated that both SO_4_˙^−^ and ˙OH were the primary reactive radicals in these systems. In addition, the degradation of RB was also significantly inhibited by the addition of trichloromethane with the efficiency decreasing to 72.9%, 19.3%, 8.5% and 5.0%, respectively. In summary, the results indicate an effect for the inhibition of RB removal, whereby the contribution rate may be ranked in the systems in the following order: SO_4_˙^−^ < O_2_˙^−^ < ˙OH.

#### 3.2.3 Role of surface metal in enhancing the reactivity of ZVI

**Fig. 6:**
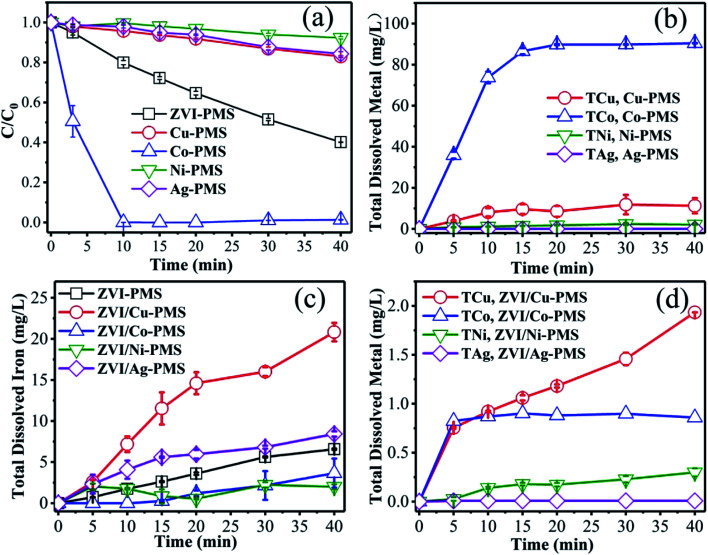
(a) RB degradation and (b) total dissolved M ions in the different systems of M/PMS (M represents Cu, Co, Ni and Ag) as a function of time. (c) TDI and (d) the total dissolved metal ions in the ZVI/M-PMS systems as a function of time. [RB]_0_ = 20 mg L^−1^, [PMS]_0_ = 1 mM, [PMS] : [RB] = 15.4 : 1, [catalyst]_0_ = 100 mg L^−1^, initial pH = 3 ± 0.2, *T* = 25 ± 1 °C.

As is known, metals can be dissolved to form homogeneous metal ions in acid solution, which can activate PMS effectively. Thus, to ascertain the contribution of Cu(ii), Co(ii), Ni(ii) and Ag(i) catalysis, the degradation experiments were performed only in the presence of PMS and Cu^0^, Co^0^, Ni^0^ and Ag^0^. As shown in Fig. 6(a), the amount of RB degraded was 17.0%, 98.6%, 7.7% and 15.7% in the presence of Cu^0^, Co^0^, Ni^0^ and Ag^0^, respectively, which showed that potential activation by the added metal ions alone was negligible, except Co. Simultaneously, as shown in Fig. 6(b), the total dissolved M ions was monitored in the same systems of Fig. 6(a) during the reaction process. After 40 min treatment, the total M concentration was 15.0 mg L^−1^, 90.4 mg L^−1^, 2.1 mg L^−1^ and 0.01 mg L^−1^ in the presence of Cu, Co, Ni and Ag, respectively, which is consistent with the removal efficiency of RB (Fig. 6(a)). Meanwhile, as can be seen in Fig. 6(c) and (d), in the effluent of the ZVI/M bimetallic system, the concentration of the total dissolved iron (TDI) and the total dissolved M ions was monitored during the reaction process. Fig. 6(c) shows that the TDI of the ZVI control experiment was higher than of the ZVI/Co and ZVI/Ni bimetallic systems, but lower than of the ZVI/Cu and ZVI/Ag bimetallic systems, and Fig. 6(d) demonstrates that the concentration of the M leaching during the process was so small that their contribution to the removal of RB was negligible except Co.

Furthermore, recently, it was reported that PMS was activated for contaminant degradation *via* a ferryl intermediate (Fe(iv)). For instance, Zhen Wang *et al.* observed that Fe(iv)-oxo species were produced in the Fe(ii)/PMS system at acidic pH.^49^ However, Fig. S9–S13† show that Fe(iv) is not the dominant reactive intermediate in our systems.

**Scheme 1:**
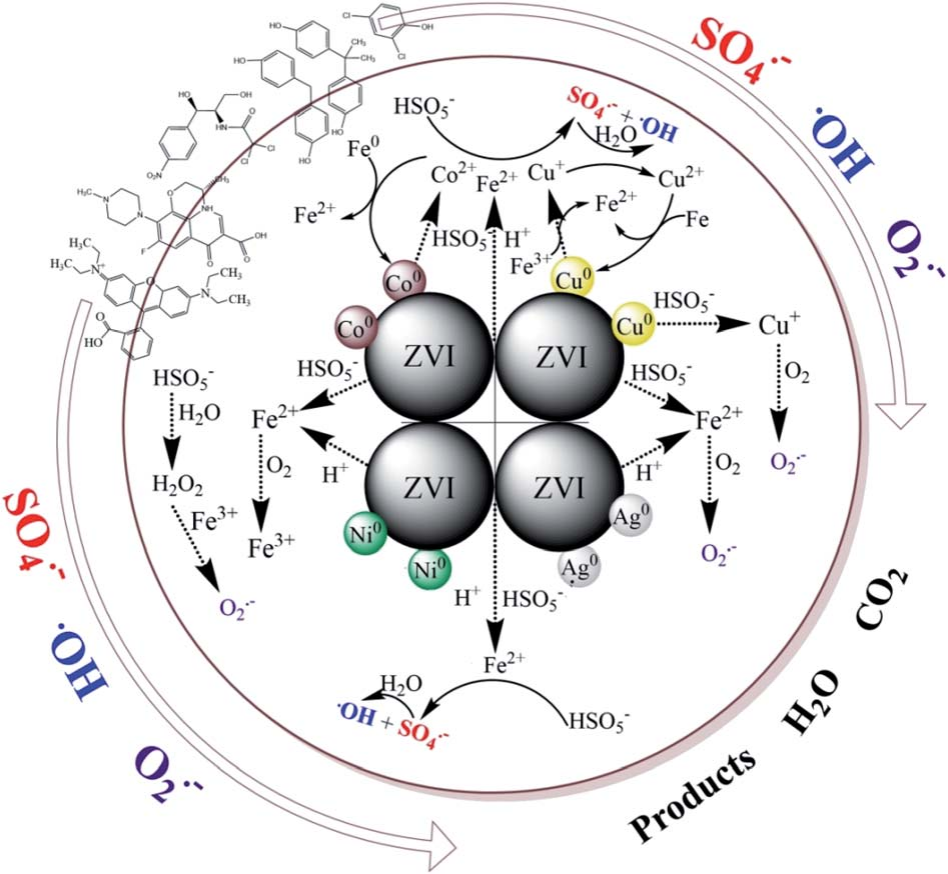
Proposed mechanism.

As presented above, there must be some interaction between M and ZVI that affects the activation of PMS, and subsequently, the degradation of contaminants. Many researchers have extensively studied iron-based bimetallic materials, which are used in the degradation of persistent pollutants.^50,51^ The mechanisms for a superior bimetallic system can be explained, (i) the formation of galvanic couples can accelerate the corrosion of iron; (ii) the second metal directly serves as a catalyst, similar to Cu in the ZVI/Cu bimetallic system; (iii) the second metal may retard the precipitation of corrosion products on the surface, and sustain the catalytic ability; (iv) the non-uniform deposition of a second metal on the surface of the iron base improves its surface roughness, which can enhance the catalytic performance of the newly formed particles. In this work, a possible mechanism for the removal of contaminants by the ZVI/M bimetallic system is proposed in Scheme 1. The primary route is that, initially, the formation of galvanic couples between ZVI and the depositing metal can affect the iron corrosion and the electron transfer from ZVI to the contaminants.^52^ Secondly, the contaminant degradation capacity of the ZVI/M bimetallic system is correlated with the corrosion of ZVI because the TDI and the total dissolved M ions can activate PMS for generating reactive radicals (˙OH, SO_4_˙^−^ and O_2_˙^−^) (eqn (1)–(12)).^8,45,46^ The ZVI/Co and ZVI/Ni bimetallic systems restrained the corrosion of ZVI and although Co leaching could activate PMS, its leaching was only 0.86 mg L^−1^ at 40 min and its effect was not sufficient to counteract Fe^2+^. In contrast, the ZVI/Cu and ZVI/Ag bimetallic systems could facilitate the corrosion of ZVI, which may be because the leached Cu and Ag could be reduced to Cu^0^ and Ag^0^, leading to the release of Fe^2+^*via*eqn (13) and (14). Moreover, the difference between the bimetallic systems is also ascribed to their potential. As shown in Fig. S14,† the potential of a glassy carbon electrode (GCE) with ZVI and bimetallic particle coatings was different. Specifically, the potential of ZVI/Ag increase to around +0.75 V, which is higher than that of ZVI (+0.70 V). Inversely, the potential of ZVI/Co and ZVI/Ni decreased significantly.^53^ For the ZVI/Cu bimetallic particles, although the GCE with the ZVI/Cu bimetallic particles exhibited a lower potential than that with ZVI, the leached Cu turned into Cu^+^ species *via*eqn (1) and (2),^29,54^ which can activate PMS to generate SO_4_˙^−^ (eqn (15)).^29^

13 Cu^2+^ + Fe^0^ → Fe^2+^ + Cu^0^

14 2Ag^+^ + Fe^0^ → Fe^2+^ + 2Ag^0^

15 Cu^+^ + HSO_5_^−^ → Cu^2+^ + SO_4_˙^−^ + OH^−^

### 3.3 Effect of bimetallic ratio on RB degradation

**Fig. 7:**
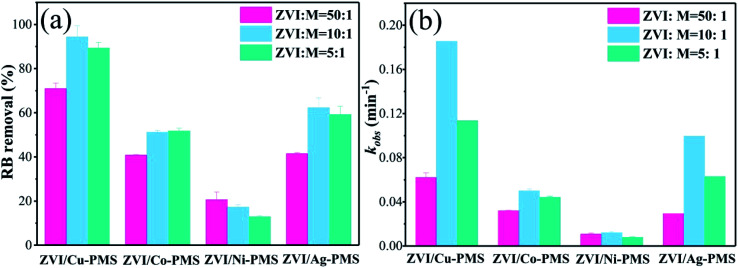
(a) RB degradation when the bimetallic ratio (M : ZVI) was 1 : 50, 1 : 10 and 1 : 5 within 20 min in the different systems; (b) reaction rates of the different systems within 20 min. [RB]_0_ = 20 mg L^−1^, [PMS]_0_ = 1 mM, [PMS] : [RB] = 15.4 : 1, [catalyst]_0_ = 100 mg L^−1^, initial pH = 3 ± 0.2, *T* = 25 ± 1 °C.

Previous studies found that the reduction of pollutants is related to the transition metal mass loading.^28^ The EDS spectra and elementary compositions of bimetallic particles are shown in Fig. S4–S7† and Table 1, respectively. Table 1 shows that the amount of Cu on the ZVI surface was much higher than that of Co, Ni and Ag, especially, the Ag mass loading could not be detected due to its super low quantity on the surface. Thus, the results suggested that the metallic Cu, Co and Ni were integrated into ZVI and the inert metal mass loading increased with an increase in the molar ratio (M : ZVI). Also, three different bimetallic ratios were used to confirm the effect of the bimetallic ratio on catalytic activity. As shown in Fig. 7, the RB removal efficiency in the ZVI/Cu-PMS system reached 99.76%, 99.78% and 99.89% when the M : Fe ratio was 1 : 50, 1 : 10 and 1 : 5, respectively. Also, the *k*_obs_ of the ZVI/Cu-PMS system was increased from 0.0709 to 0.1949 min^−1^ but then decreased to 0.0977 min^−1^ with an further increase in the M : ZVI ratio (Fig. S15†), which may be related to the full coverage of Cu on the surface of ZVI, inhibiting the activity of the bimetallic catalyst. The ZVI/Co-PMS system showed an increase in the RB degradation efficiency (72.01%, 86.42%, 82.85%) and reaction rate (0.0289, 0.0414 and 0.0431 min^−1^) with an increase in the M : ZVI ratio from 1 : 50 to 1 : 5.

The release of Fe^2+^ was accelerated using the bimetallic particles compared to ZVI because of the galvanic reaction and replacement reaction. With an increase in the metal ratio, the layer of inert metal (Cu/Co/Ni/Ag) on the surface of ZVI became uniform and fully covered its surface. When the M : ZVI ratio was higher than a certain range, less ZVI/M cells were formed and the electron transfer rate decreased. Consequently, the degradation rate of RB also decreased.^27,31^ In addition, the inert metal wrapped ZVI completely, which prevented the contact of ZVI and H^+^, and hence reduced the corrosion of ZVI.

### 3.4 Effect of catalyst dosage on RB degradation

**Fig. 8:**
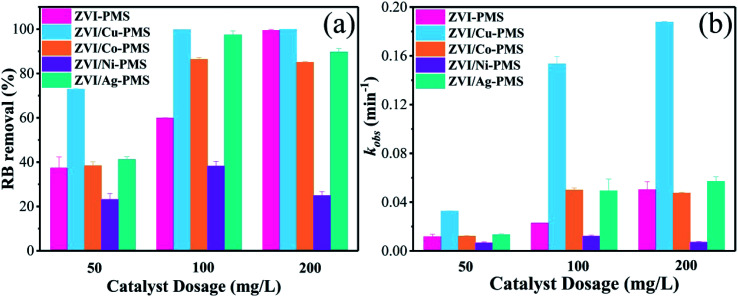
(a) RB removal ratio and (b) *k*_obs_ with the different catalysts of different dosages. [RB]_0_ = 20 mg L^−1^, [PMS]_0_ = 1 mM, [PMS] : [RB] = 15.4 : 1, [catalyst]_0_ = 50, 100, 200 mg L^−1^, initial pH = 3 ± 0.2, *T* = 25 ± 1 °C.

The decomposition performance of the system was dominated by the quantity of free ferrous ions, which played a key role in the activation of PMS (eqn (10) and (11)). Previous studies showed that when the catalysts dosage increased, the surface area and active catalytic sites also increased, and more galvanic couples formed, resulting in an enhancement in the pollutant removal efficiency.^27^ Therefore, the effect of catalyst dosage on the removal of RB was further investigated, and the final degradation efficiencies of RB within 40 min and the pseudo-first-order kinetic parameters (*k*_obs_) are shown in Fig. 8. As can be seen, the RB removal efficiency increased from 72.59% to 99.78% with an increase in the ZVI/Cu catalyst dosage from 50 to 100 mg L^−1^, meanwhile, the *k*_obs_ increased to 0.1949 min^−1^ at 100 mg L^−1^, which is 6.72 times faster than that (0.0290 min^−1^) at 50 mg L^−1^. However, only a slight enhancement in the RB removal efficiency and *k*_obs_ when the dosage increased from 100 to 200 mg L^−1^. The ZVI, ZVI/Co, ZVI/Ni and ZVI/Ag catalysts showed the same tendency as ZVI/Cu. The XPS spectra in Fig. 3 verify these phenomena, where the surface of the catalysts was covered with a thin oxide layer, which may mitigate the release of Fe^2+^ and the galvanic reaction.

### 3.5 Effect of initial pH on RB degradation

**Fig. 9:**
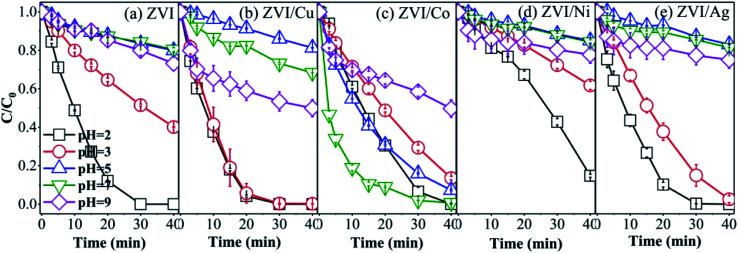
RB degradation in the different systems ((a) ZVI-PMS system, (b) ZVI/Cu-PMS system, (c) ZVI/Co-PMS system, (d) ZVI/Ni-PMS system, and (e) ZVI/Ag-PMS system) with the initial pH value of 2, 3, 5, 7 and 9. [RB]_0_ = 20 mg L^−1^, [PMS]_0_ = 1 mM, [PMS] : [RB] = 15.4 : 1, [catalyst]_0_ = 100 mg L^−1^, initial pH = 2, 3, 5, 7, 9 ± 0.2, *T* = 25 ± 1 °C.

The five catalysts were used to activate PMS to degrade RB with different initial pH, and the results are shown in Fig. 9. The ZVI, ZVI/Cu and ZVI/Ag catalysts showed the best catalytic ability under extremely acidic conditions, where RB was completely removed at the pH value of 2, and with an increase in the pH value, the RB degradation efficiency obviously decreased to 21.35%, 18.83% and 13.59%, respectively. This phenomenon can be explained by the fact that the hydroxides on the surface of the catalysts could not be dissolved easily at high pH conditions, and the amount of free iron species decreased with an increase in pH. For example, when the pH value was 5, the maximum Fe^3+^ concentration was only 4 × 10^−11^ mol L^−1^ (*K*_sp_ (Fe(OH)_3_) = 4 × 10^−38^ mol L^−1^).

The ZVI/Co catalyst showed the highest RB removal ratio (99.33%) and the fastest reaction rate (0.1302 min^−1^) at the initial pH of 7, which agrees with previous studies.^55^ In the ZVI/Co-PMS system, the OH^−^/H_2_O would be oxidized into ˙OH by SO_4_˙^−^ and HSO_5_^−^ at neutral pH (eqn (10)–(12)). The presence of both ˙OH and SO_4_˙^−^ (dominator) maintains a relatively high decontamination efficiency.^56^

As shown in Fig. 9(e), the RB removal efficiency was relatively enhanced at pH 9 compared with neutral condition in the ZVI-PMS, ZVI/Cu-PMS and ZVI/Ag-PMS systems, which illustrates that PMS can also be activated by alkali.^13^

### 3.6 Experiments in various water matrices

**Fig. 10:**
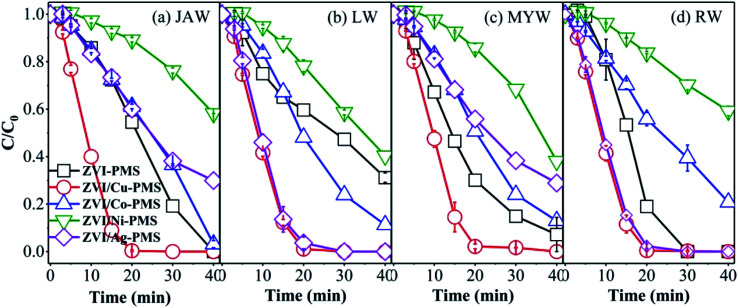
RB degradation in the different systems with various water matrices of (a) JAW, (b) LW, (c) MYW and (d) RW. [RB]_0_ = 20 mg L^−1^, [PMS]_0_ = 1 mM, [PMS] : [RB] = 15.4 : 1, [catalyst]_0_ = 100 mg L^−1^, initial pH = 3 ± 0.2, *T* = 25 ± 1 °C.

To examine real conditions, experiments with various raw water samples on the removal of RB were performed using the different systems. The raw water samples were collected at Sichuan University. The characteristics of the various raw water samples are displayed in Table S2,† which show a near neutral pH and the dissolved organic matter and the inorganic species existing in the various water matrices. Some previous studies demonstrated that the raw water can affect the effectiveness of the oxidation process.^48,57^ However, as shown in Fig. 10, in the ZVI/Cu-PMS and ZVI/Ag-PMS systems, the degradation efficiency of RB was nearly 100% within 40 min reaction in the river water named Jiang'an (JAW), landscape water (LW), Mingyuan lake water (MYW) and running water (RW), while the degradation efficiency in deionized water (DW) was 99.78% in ZVI/Cu-PMS and 97.43% in ZVI/Ag-PMS, respectively. Similarly, compared with DW, the removal efficiency of RB was negligible in the various raw water samples. In general, apparently, the five real water samples showed an enhanced effect on RB removal and the ZVI/Cu-PMS system illustrated the best potential to treat RB wastewater.

## 4. Conclusion

This work showed that bimetallic particles (ZVI/Cu and ZVI/Ag) resulted in a significant improvement to active PMS in the degradation of contaminants compared to ZVI, but the ZVI/Co and ZVI/Ni bimetallic systems restrained the degradation of contaminants. The mechanism investigation revealed that iron leaching was the main contributor for PMS activation in our systems, and the formation of galvanic couples between ZVI and the deposited metal of Cu or Ag could accelerate the iron corrosion, but the ZVI/Co and ZVI/Ni bimetallic systems restrained the iron corrosion. The EPR analysis and radical quenching tests revealed that reactive radicals (˙OH, SO_4_˙^−^ and O_2_˙^−^) were responsible for the degradation of contaminants. Also, the key operating parameters were optimized by batch experiments, which showed that the removal of contaminants increased with an increase in the metal ratio (M : ZVI) to a certain value, higher bimetal catalyst dosage and lower pH (except for ZVI/Co). It is worth noting that the effect of five water matrices on the removal efficiency of RB was negligible, which proves that the bimetallic particles (especially ZVI/Cu and ZVI/Ag) have great potential for the treatment of real wastewater.

## Conflicts of interest

There are no conflicts to declare.

## Supplementary Material

RA-010-D0RA03924A-s001
