# Supplementary material for: Removal of contaminants by activating peroxymonosulfate (PMS) using zero valent iron (ZVI)-based bimetallic particles (ZVI/Cu, ZVI/Co, ZVI/Ni, and ZVI/Ag)
Source: RSC Adv. 2020 Jul 28;10(47):28232–42. doi: 10.1039/d0ra03924a (PMC9055671; doi:10.1039/d0ra03924a)
Supplement: RA-010-D0RA03924A-s001 [file RA-010-D0RA03924A-s001.pdf]

Electronic Supplementary Material (ESI) for RSC Advances.
This journal is © The Royal Society of Chemistry 2020

Supplementary Materials

for

Removal of contaminants by activating peroxymonosulfate using zero valent iron based bimetallic particles (Fe/Cu, Fe/Co, Fe/Ni, Fe/Ag)

Xiaowei Huo[a], Peng Zhou[a], Yunxin Liu[a], Feng Cheng[a], Yang Liu[a], Xin Cheng[a, b], Yongli Zhang*[, a], Qingguo Wang*[, a]

[a] *College of Architecture & Environment, Sichuan University, Chengdu 610065, PR China*

[b] *Department of Chemical and Environmental Engineering, Yale University, New Haven, Connecticut 06511, United States*

* Corresponding author

E-mail address: xyl_scu@126.com (Y. Zhang), wangqgscu@163.com (Q. Wang)

Test S1

Micron zero valent iron powder (ZVI, Fe^0, ≥ 99.9 %), Micron zero vatent copper powder (Cu^0, ≥ 99.9 %), Micron zero vatent cobalt powder (Co^0, ≥ 99.9 %), Micron zero vatent nickel powder (Ni^0, ≥ 99.5 %), Micron zero vatent silver powder (Ag^0, ≥ 99.9 %), 2,4-dichlorophenol (2,4-DCP, ≥ 99 %), bisphenol A (BPA), bisphenol F (BPF), levofloxacin (LFX), chloramphenicol (CAP), methanol (HPLC grade) and acetonitrile (HPLC grade) were purchased from Aladdin Industrial Corporation. Oxone (PMS, $KHSO_5 \cdot 0.5KHSO_4 \cdot 0.5K_2SO_4$, ≥ 99.5 %) was purchased from Sigma-Aldrich. Methyl phenyl sulfoxide (PMSO, ≥ 98.0 %), methyl phenyl sulfone ($PMSO_2$, ≥ 98.0 %) were purchased from Shanghai Macklin Biochemical Co., Ltd. Rhodamine B (RB, ≥ 99%), copper sulfate, cobaltous sulfate, nickel sulfate, silver nitrate, formic acid, sulfuric acid, sodium hydroxide, ethanol, tert-butyl alcohol, phenol, nitrobenzene (NB) and sodium nitrite were all supplied by Chengdu Kelong chemical reagent factory.

Test S2

The HPLC chromatography (Waters, e2695) was equipped with a 2489 UV-vis detector and the detection wavelength and binary phase composition were as follows: 286 nm and methanol/0.1% formic acid (70:30, v/v) for 2,4-DCP; 276 nm and methanol/0.1 % formic acid (70:30, v/v) for BPA; 230 nm and methanol/0.1 % formic acid (65:35, v/v) for BPF; 293 nm and acetonitrile /0.1 % formic acid (13:87, v/v) for LFX and 277 nm and acetonitrile /0.1 % formic acid (30:70, v/v) for CAP. The flow rate of these binary phases was 1 mL/min and the temperature of liquid chromatographic column was 35 ^oC.

Test S3

The bimetallic particles' surface topography and chemical composition before and after reaction were characterized by scanning electron microscopy (SEM, JSM-7500F (JEOL, Japan)) and X-ray diffraction (XRD, X'Pert Pro MPD diffractometer (Panalytical, Holland)), respectively. The surface element distribution and valence state of the pristine bimetallic particles were characterized by energy dispersive spectrometer (EDS, JSM-7500F (JEOL, Japan)) and X-ray photoelectron spectroscopy (XPS, AXIS Ultra DLD (Kratos, Britain)).

Test S4

The EPR spectrum was measured under the following conditions: a center field of 3505.40 G; a sweep width of 150.00 G, a microwave frequency of 9.84 GHz, a microwave attenuator of 20.00 dB, a microwave power of 2.00 mW, and a sweep time of 10.53 seconds.

Test S5

In this study, the existence of $PMSO_2$ were examined with ultrahigh performance liquid chromatograph (Shimazu, LC-30AD) coupled with a SCIEX TRIPLE QUAD 4500 mass spectrometry in a negative electrospray ionization mode (UPLC/ESI-MS/MS). A mixture of (A) ultrapure water and (B) acetonitrile were used as mobile phases in a binary gradient with a flow rate of 0.3 mL/min. The gradient was set as follows: B increased from 10 % to 90 % within 1.5 min and remained unchanged for 1.5 min. Then, a decreased from 90 % to 10 % within 0.1 min. Finally, it stayed for 1.9 min at the current rate. Sample injection volume was 1uL. The MS parameters were set as follows: ionspray voltage = -4500 V; source temperature = 500 °C; collision energy (CE) for ion fragments of *m/z* 79.1 was -22 V, and for ion fragments of *m/z* 63.5 was -41 V; declustering potential (DP) for ion fragments of *m/z* 79.1 was -44 V, and for ion fragments of *m/z* 63.5 was -53 V.

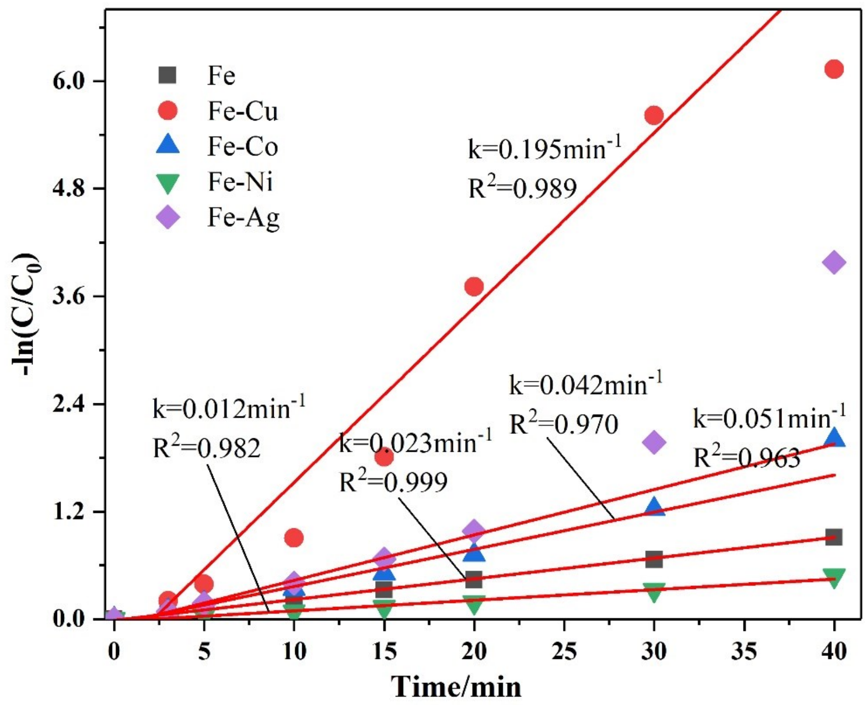

Fig. S1. The reaction rates of different systems (M: Fe = 1: 10). $[RB]_0$ = 20 mg L^{-1}, $[PMS]_0$ = 1 mM, [PMS]: [RB] =15.4:1, $[catalyst]_0$ = 100 mg L^{-1}, initial pH = 3 ± 0.2, T = 25±1 ^{o}C.

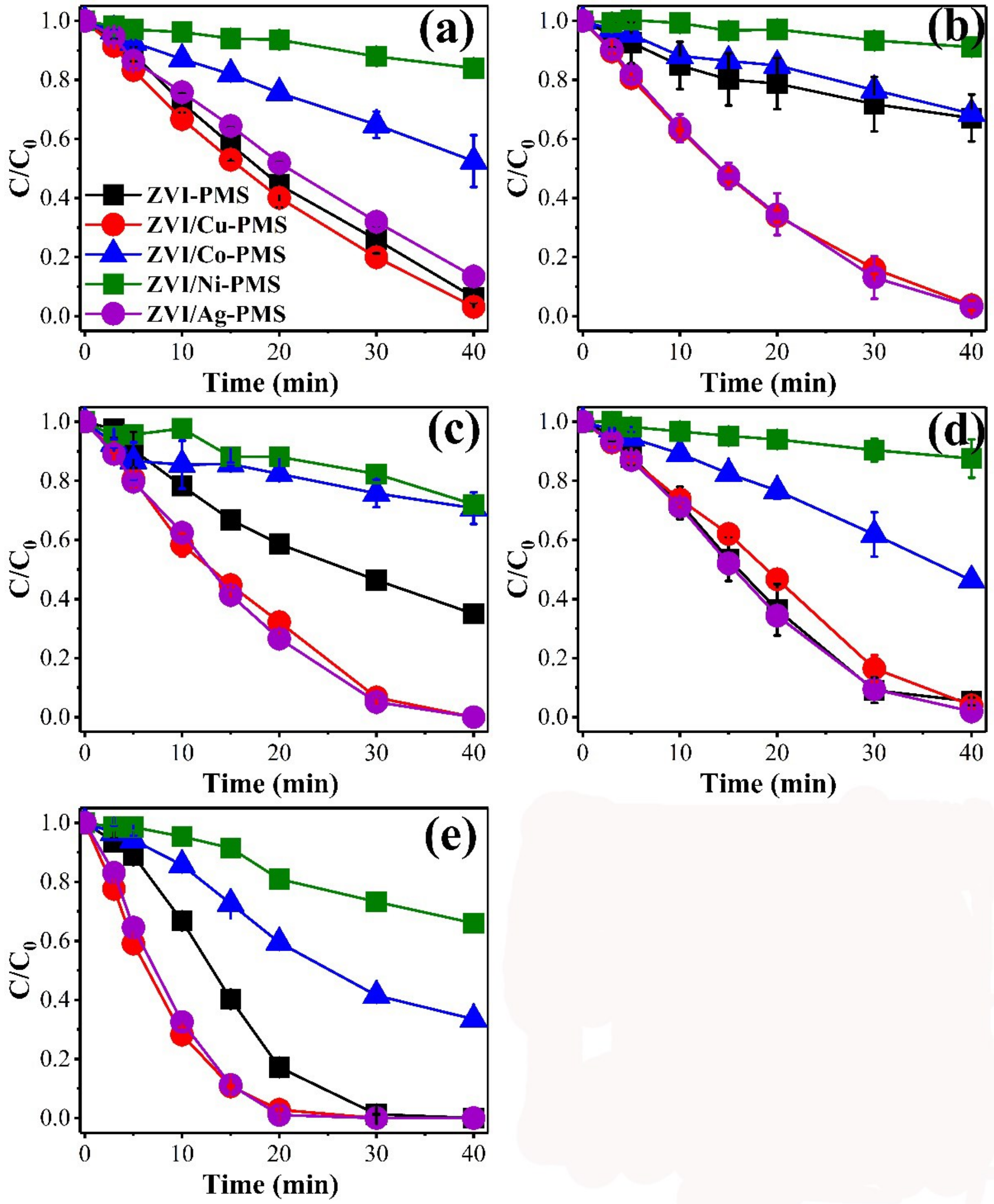

Fig. S2. Various organics compounds degradation in different systems. (a) $[2,4\text{-DCP}]_0$ = (b) $[BPA]_0$ = (c) $[BPF]_0$ = (d) $[CAP]_0$ = (e) $[LFX]_0$ = 20 mg L^{-1}, $[PMS]_0$ = 1 mM, [PMS]: [Organic contaminations] =15.4:1, $[catalyst]_0$ = 100 mg L^{-1}, initial pH = 3 ± 0.2, T = 25±1 ^oC.

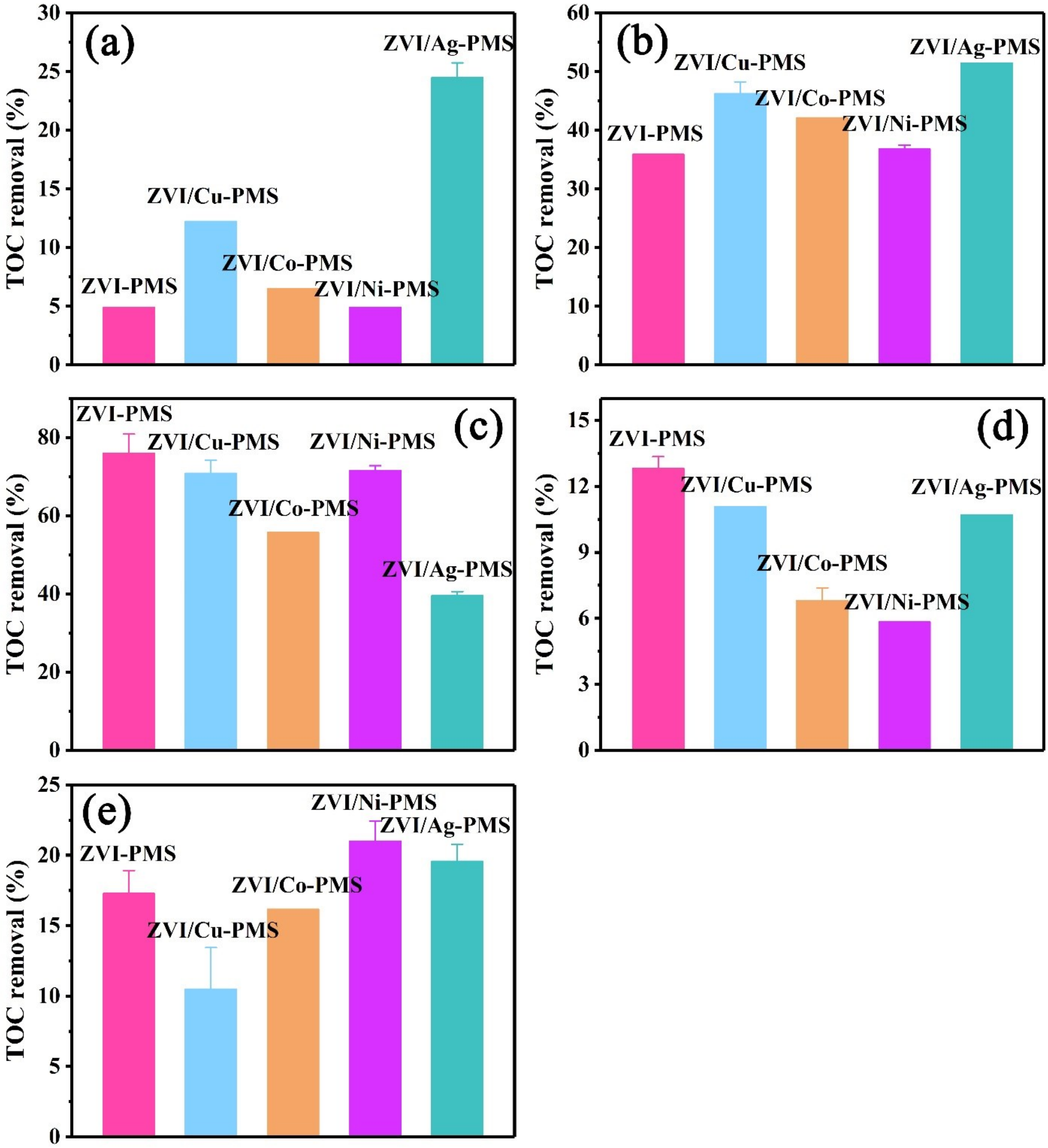

Fig. S3. The TOC (reaction time 3 h) of various organics compounds degradation in different systems. (a) [2,4-DCP]$_0$ = (b) [BPA]$_0$ = (c) [BPF]$_0$ = (d) [CAP]$_0$ = (e) [LFX]$_0$ = 20 mg L^{-1}, [PMS]$_0$ = 1 mM, [PMS]: [Organic contaminations] =15.4:1, [catalyst]$_0$ = 100 mg L^{-1}, initial pH = 3 ± 0.2, T = 25±1 ^{o}C.

Table. S1. Characteristics of various organics.

Organics	Abbreviations	Relative molecular mass	Structural formula	Chemical formula
Rhodamine B	RB	479.01	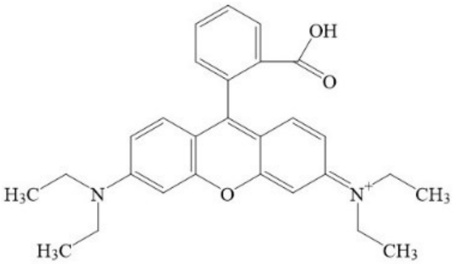	$C_{28}H_{31}ClN_2O_3$
2, 4-dichlorophenol	2,4-DCP	163.00	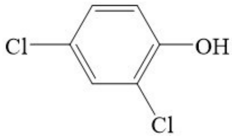	$C_6H_4Cl_2O$
Bisphenol A	BPA	228.29	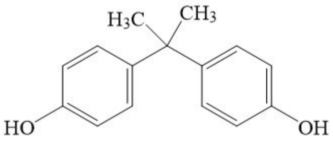	$C_{15}H_{16}O_2$
Bisphenol F	BPF	200.24	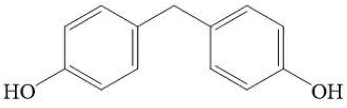	$C_{13}H_{12}O_2$
Chloramphenicol	CLP	323.14	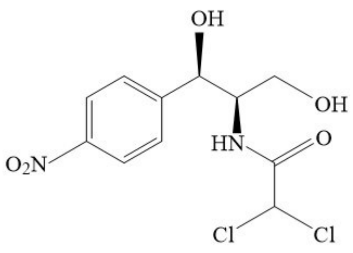	$C_{11}H_{12}Cl_2N_2O_5$

Levofloxacin	LFX	370.38	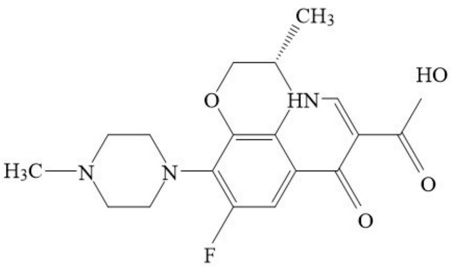	$C_{18}H_{20}FN_3O_4$

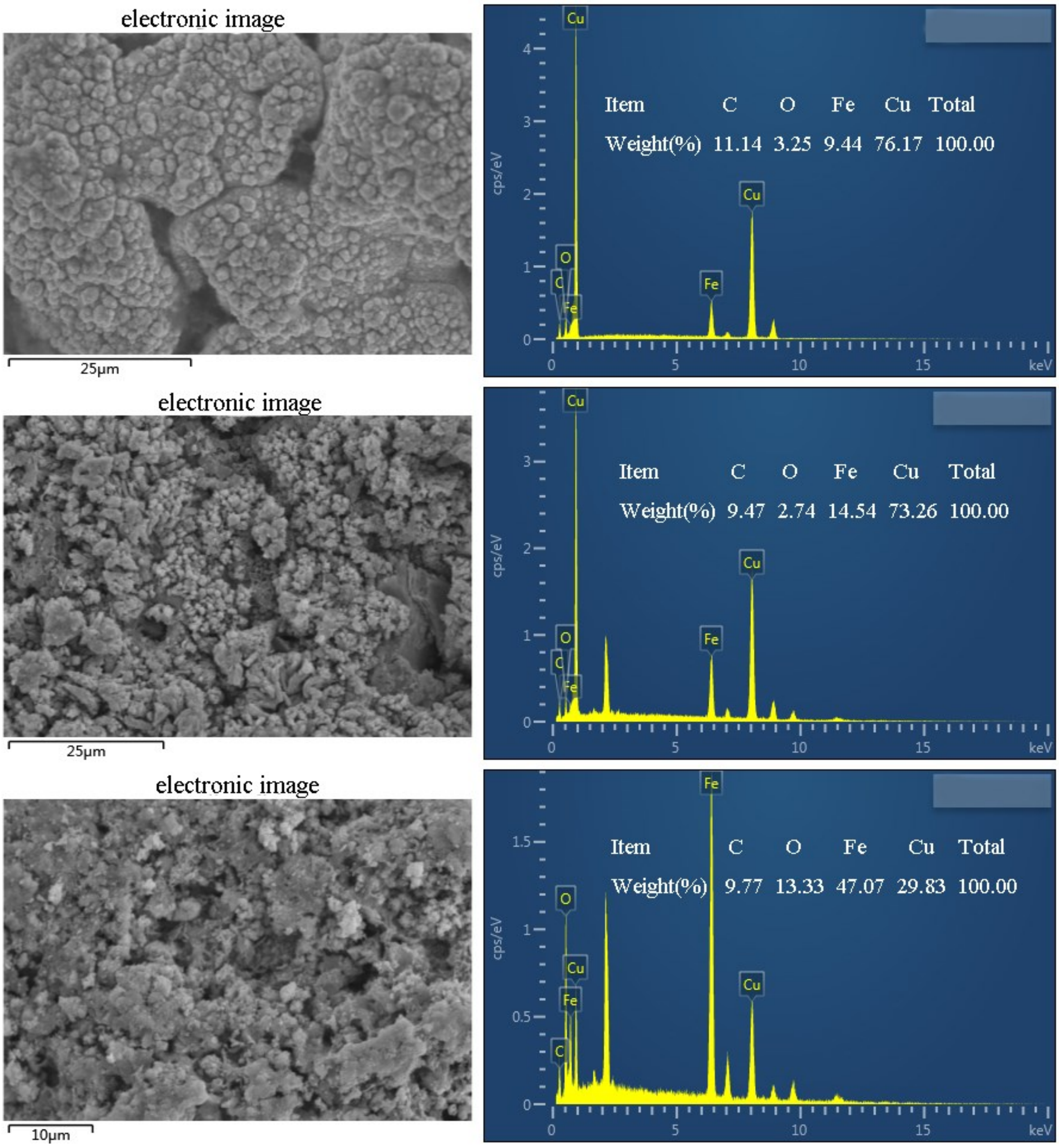

Fig. S4 SEM and EDS spectra of Fe/Cu bimetallic particles with the molar ratio (Cu: Fe) is 1: 5, 1: 10 and 1: 50.

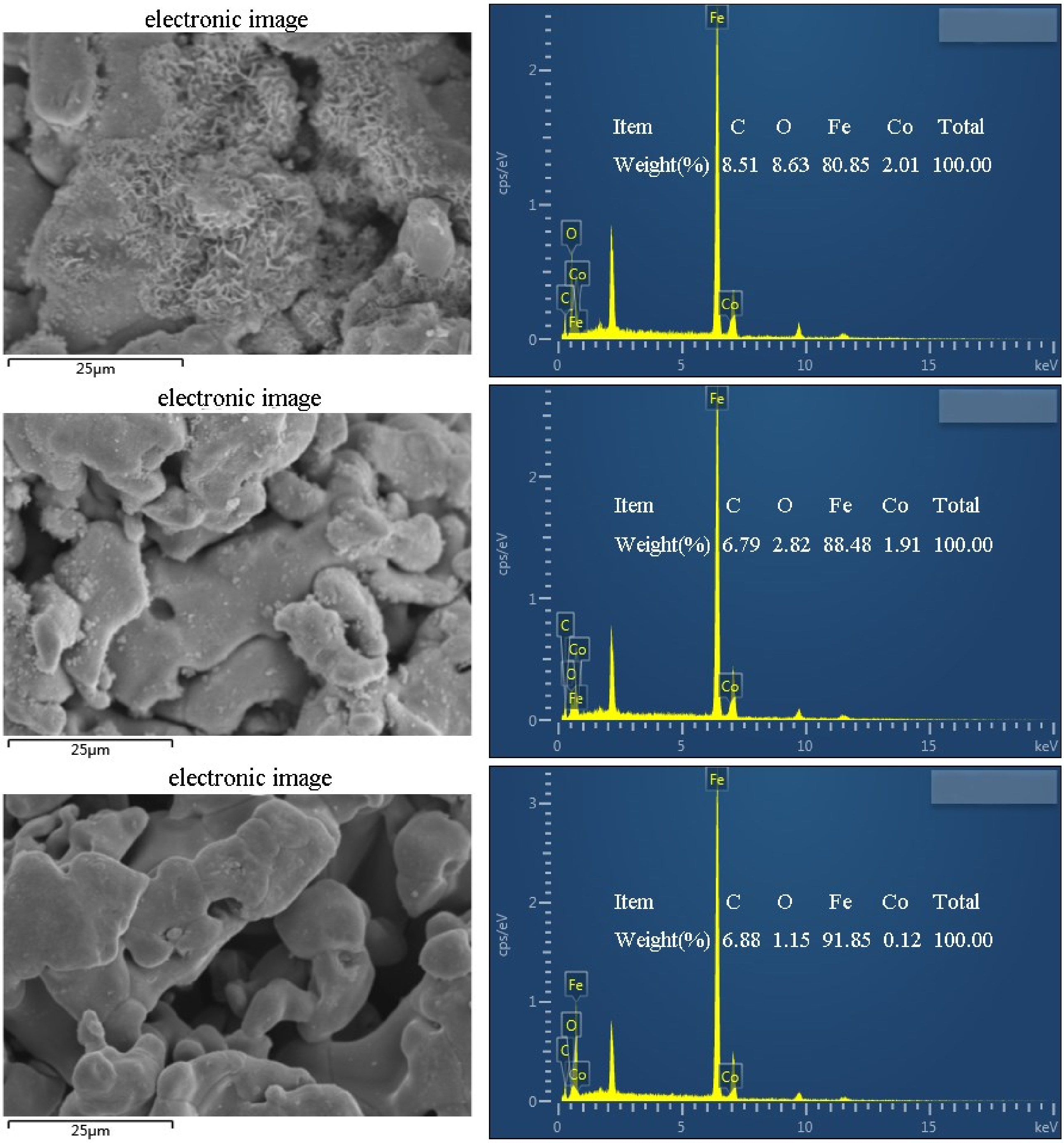

Fig. S5 SEM and EDS spectra of Fe/Co bimetallic particles with the molar ratio (Co: Fe) is 1: 5, 1: 10 and 1: 50.

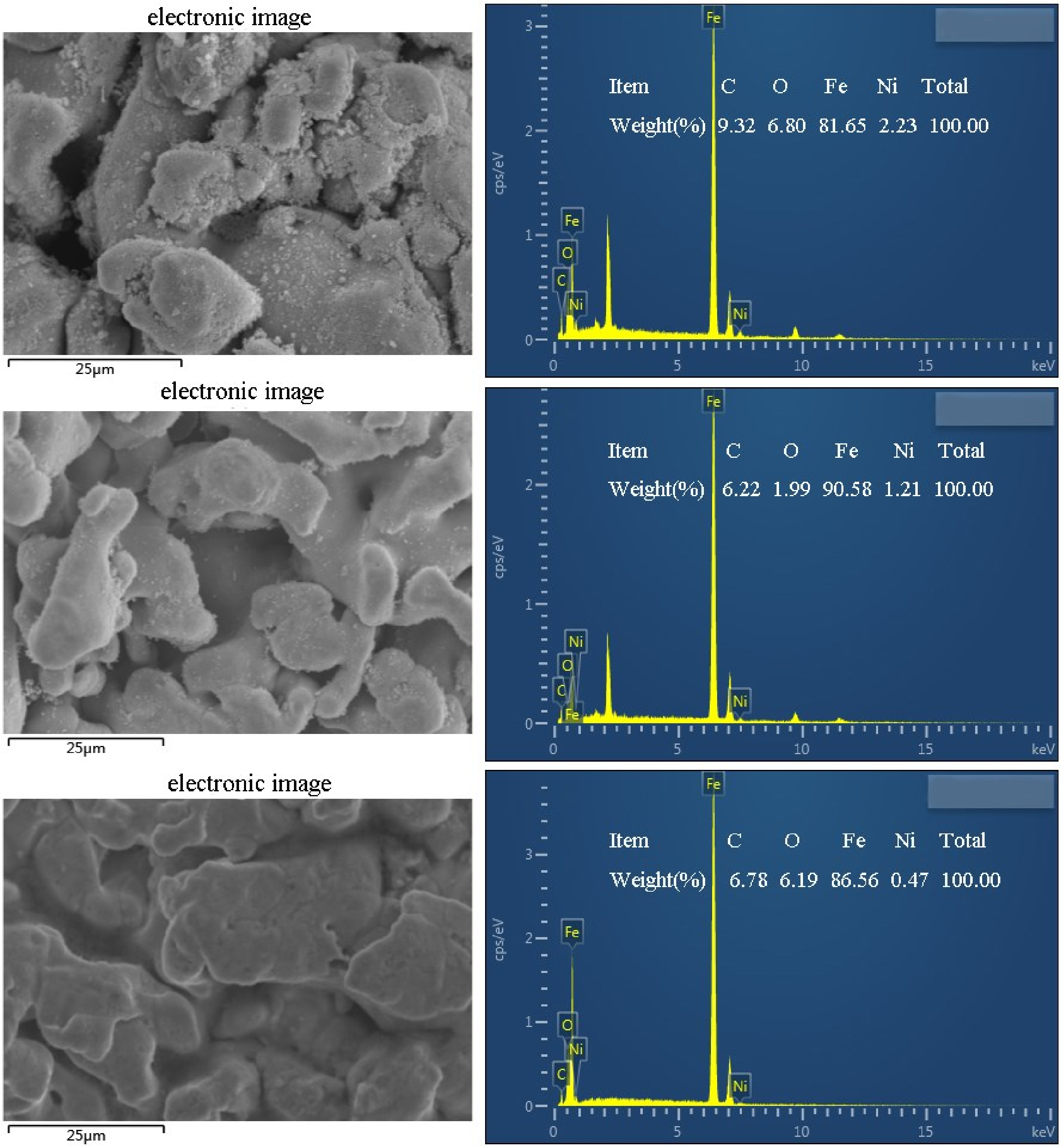

Fig. S6 SEM and EDS spectra of Fe/Ni bimetallic particles with the molar ratio (Ni: Fe) is 1: 5, 1: 10 and 1: 50.

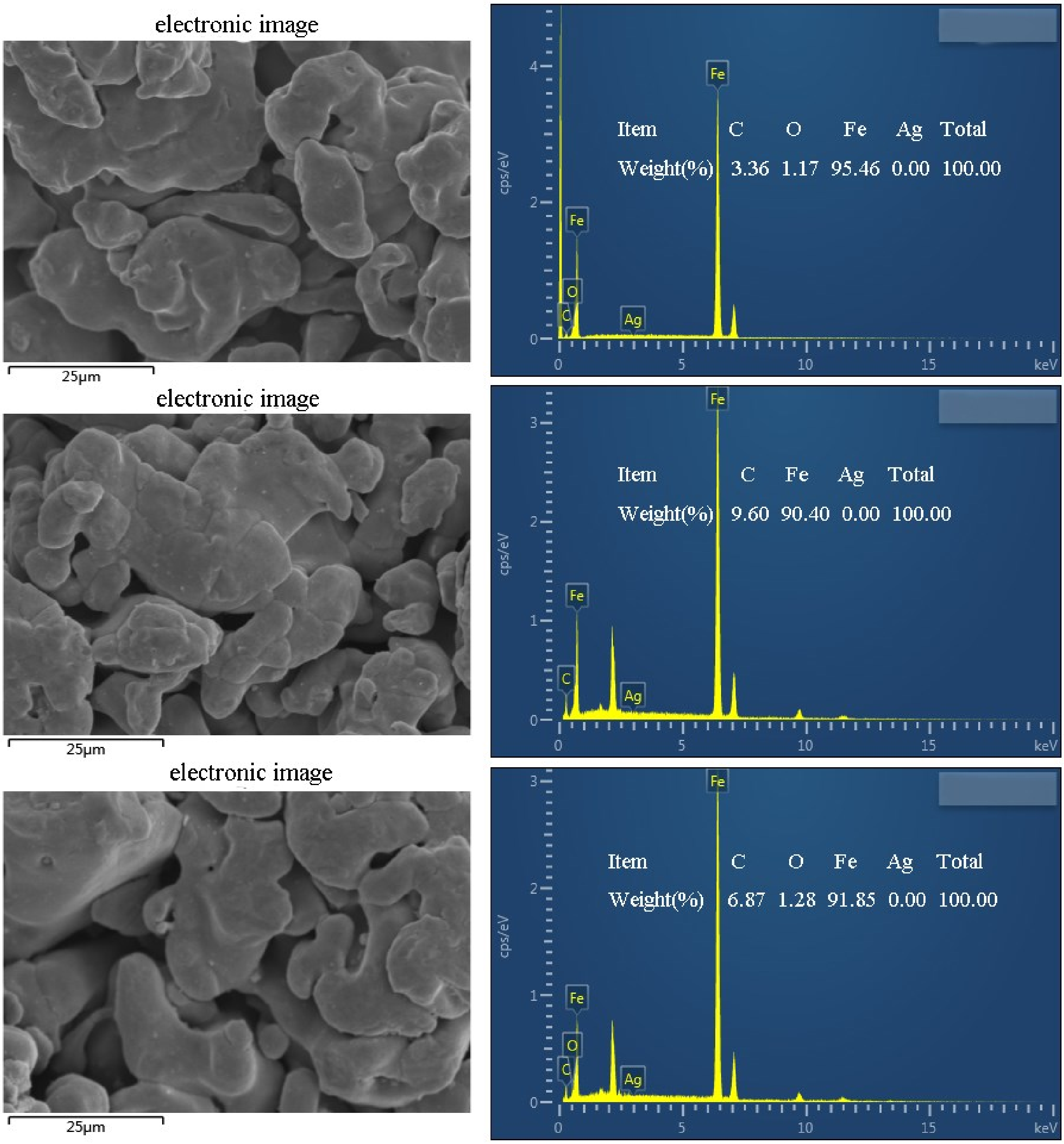

Fig. S7 SEM and EDS spectra of Fe/Ag bimetallic particles with the molar ratio (Ag: Fe) is 1: 5, 1: 10 and 1: 50.

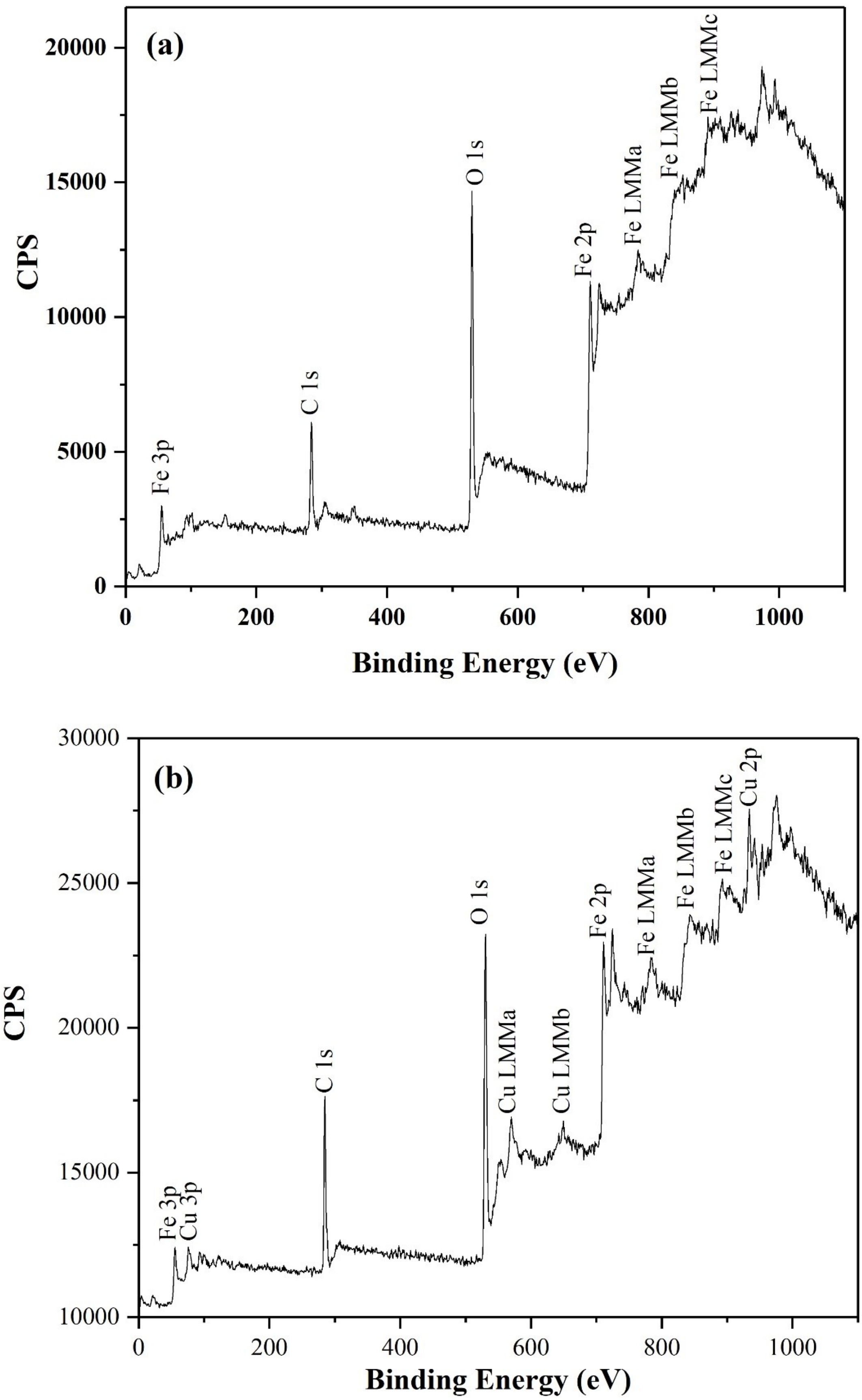
(a)
CPS
Binding Energy (eV)
Fe 3p
C 1s
O 1s
Fe 2p
Fe LMMa
Fe LMMb
Fe LMMc
(b)
CPS
Binding Energy (eV)
Fe 3p
Cu 3p
C 1s
O 1s
Cu LMMa
Cu LMMb
Fe 2p
Fe LMMa
Fe LMMb
Fe LMMc
Cu 2p

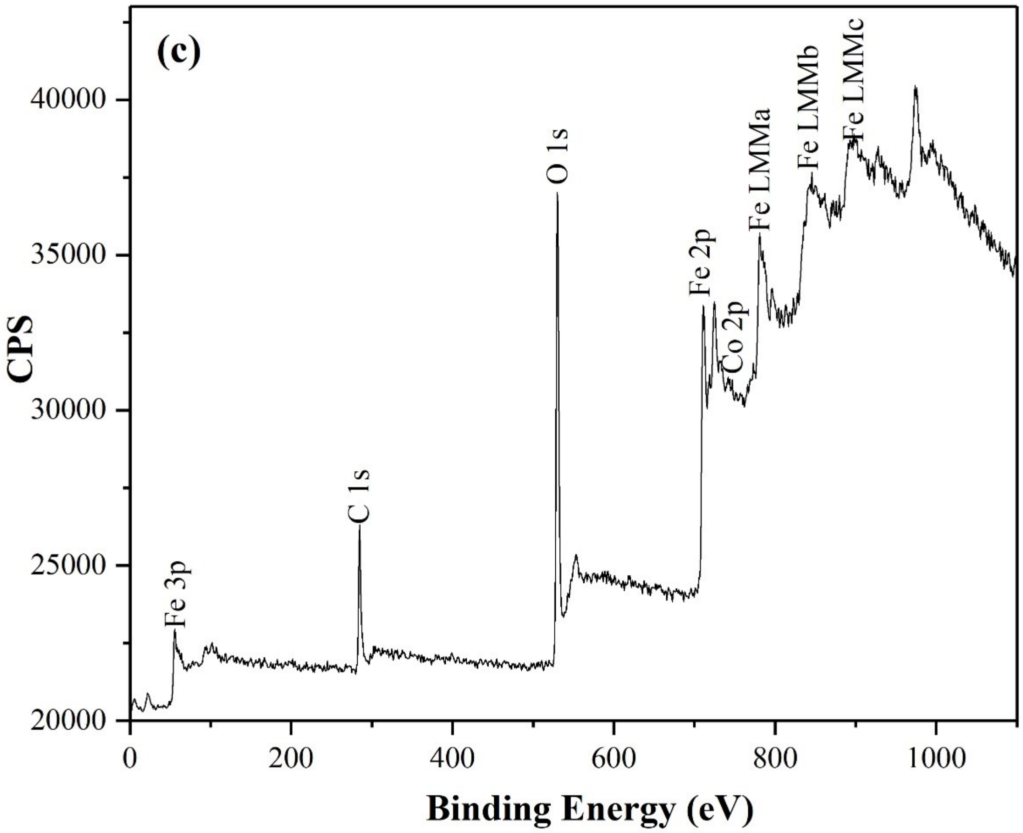
(c)
CPS
Binding Energy (eV)
40000
35000
30000
25000
20000
0
200
400
600
800
1000
Fe 3p
C 1s
O 1s
Fe 2p
Co 2p
Fe LMMa
Fe LMMb
Fe LMMc

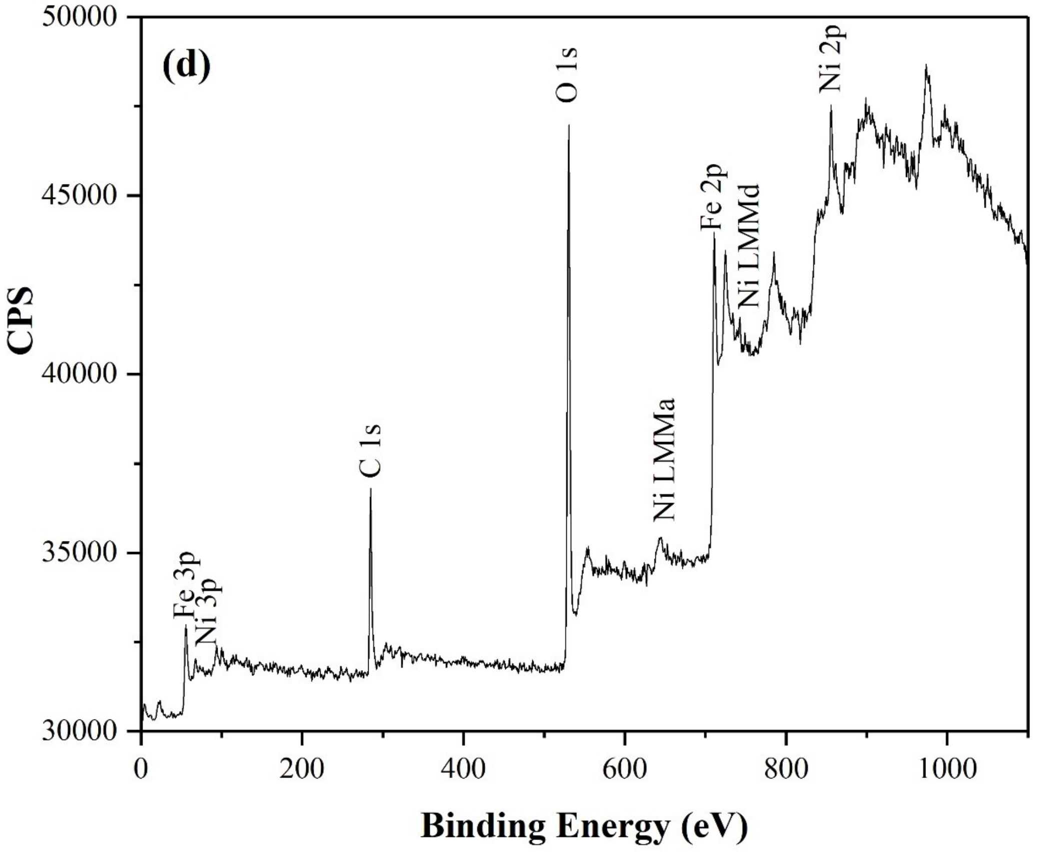
(d)
CPS
Binding Energy (eV)
50000
45000
40000
35000
30000
0
200
400
600
800
1000
Fe 3p
Ni 3p
C 1s
O 1s
Ni LMMa
Fe 2p
Ni LMMd
Ni 2p

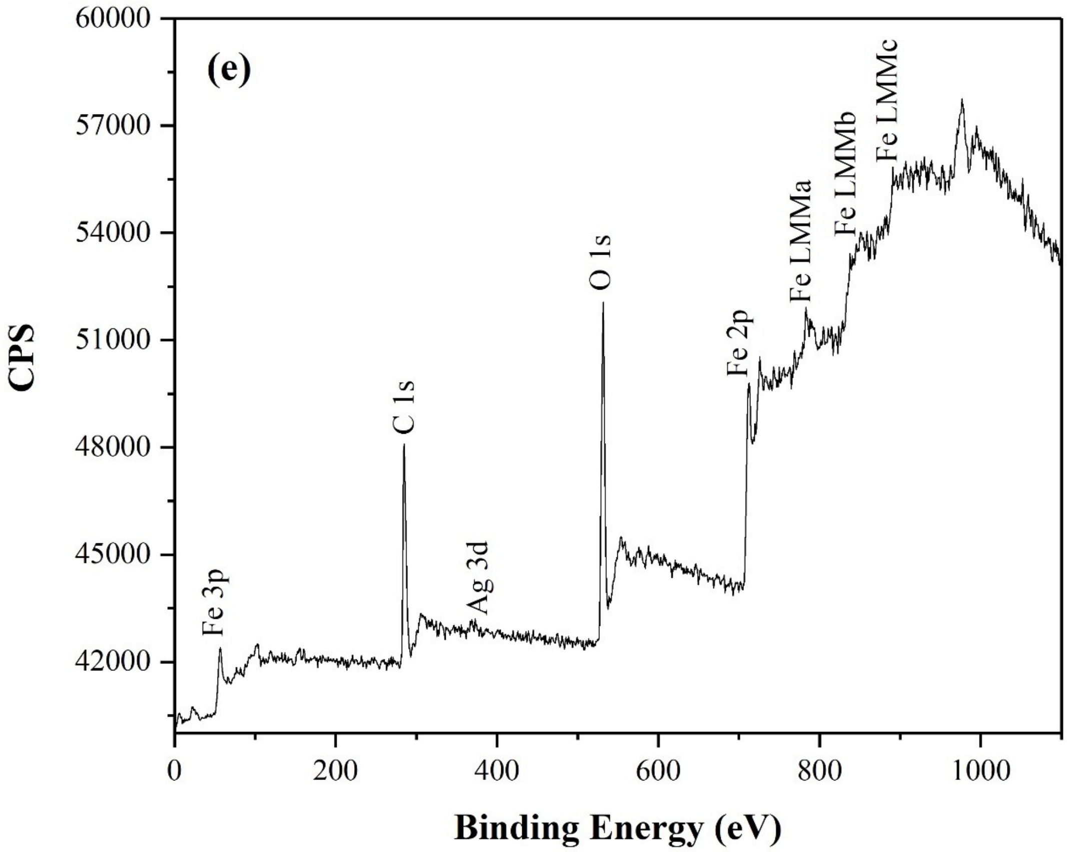

Fig. S8. XPS full-scan spectra of (a) Fe, (b) Fe/Cu, (c) Fe/Co, (d) Fe/Ni and (e) Fe/Ag.

Test S6

As we all know, Fe(IV) could oxidize PMSO to its oxygen transfer product $PMSO_2$ [1]. As shown in Fig. S9 to Fig. S12, compared with the UPLC/ESI-MS/MS chromatograms of standard $PMSO_2$ (Fig. S8), the unlabled $PMSO_2$ by oxidizing PMSO were all observed in our four systems and the intensities of the four unlabled $PMSO_2$ were practically little difference. In principle, if Fe(IV) was the dominant reactive species in our four systems, then, the removal ratio of contaminants should be similar, nevertheless, it's worth noting that the contaminants degradation efficiency in four systems were almost completely different. So according to the above results, during the process, the iron leaching was the main contributor for PMS activation in our systems and the reactive radicals ($^{\bullet}OH$, $SO_4^{\bullet-}$ and $O_2^{\bullet-}$) are possible the main reactive species.

$HSO_5^{-} + Fe^{2+} \rightarrow SO_4^{2-} + Fe^{IV}O^{2+} + H^{+}$ (S1)

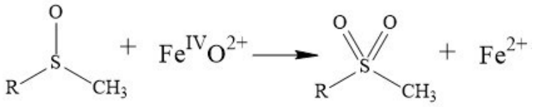

(S2)

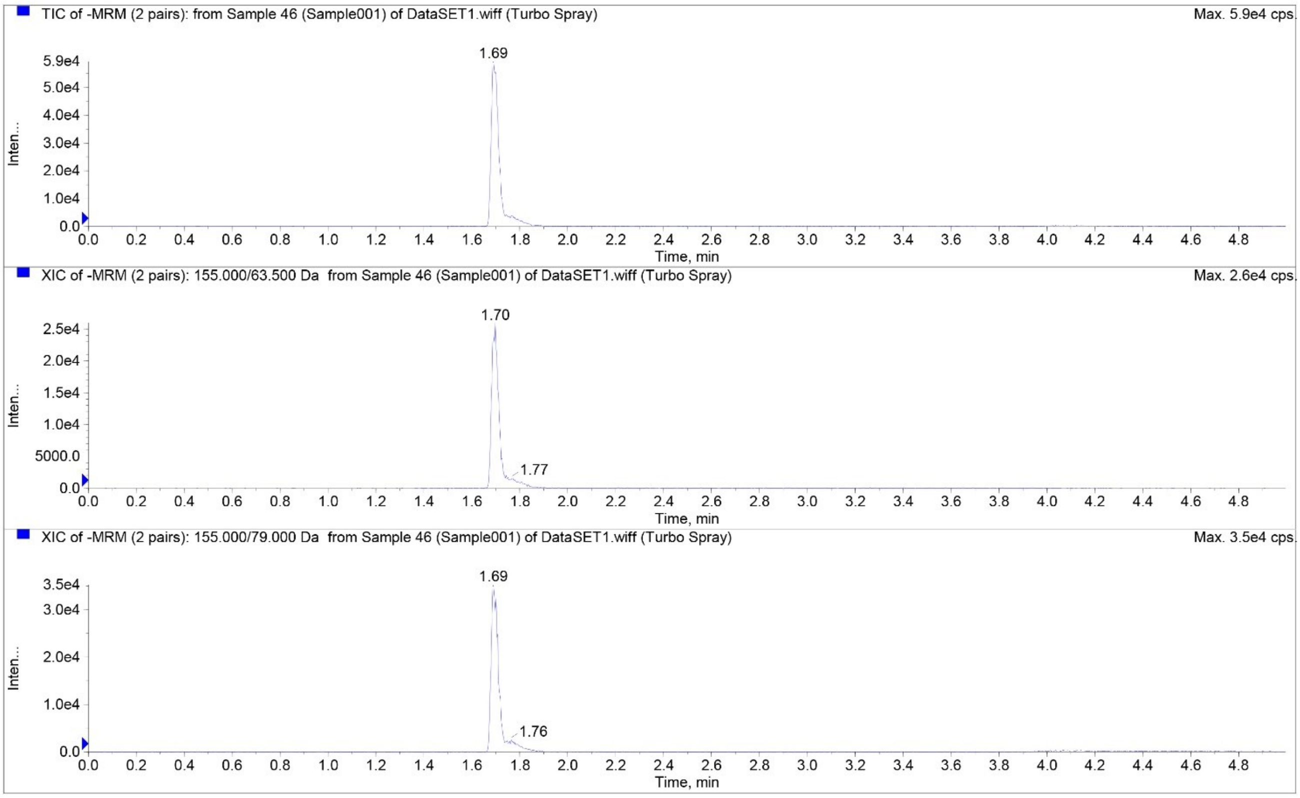

Fig. S9. UPLC/ESI-MS/MS chromatograms of standard $PMSO_2$.

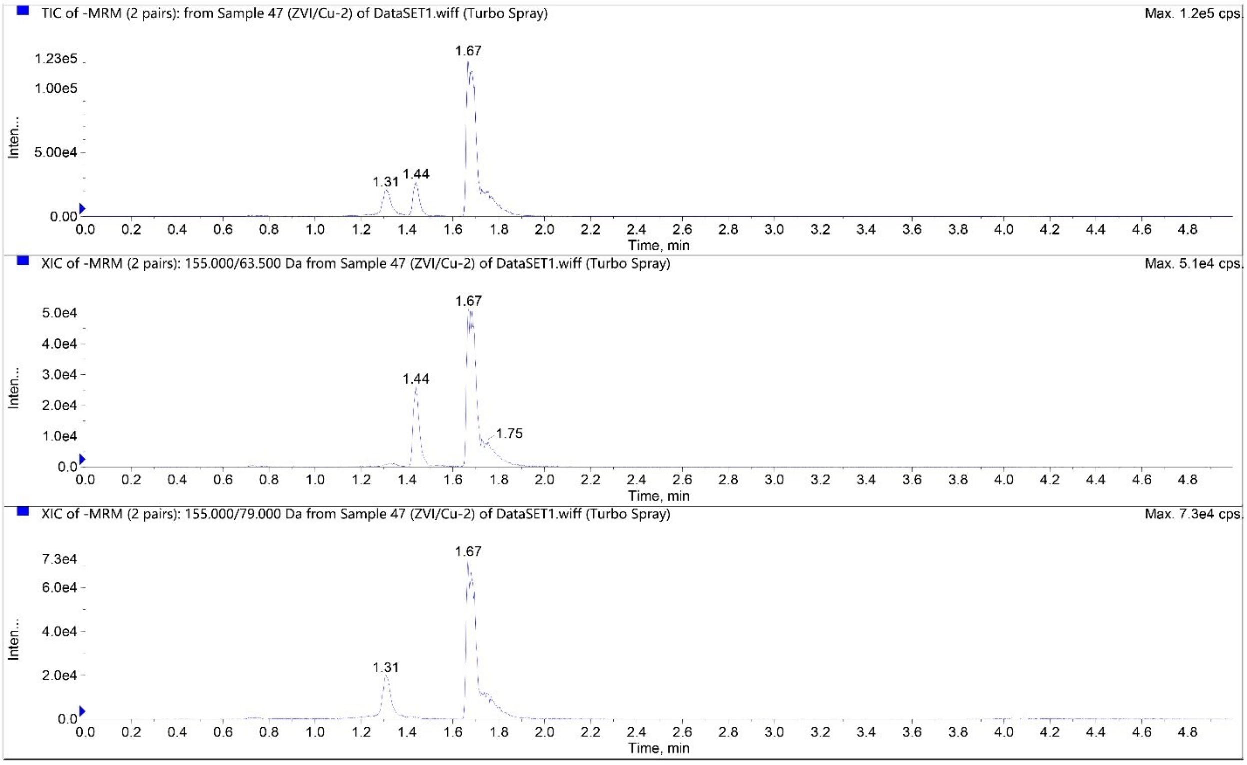

Fig. S10. UPLC/ESI-MS/MS chromatograms of unlabled $PMSO_2$ produced by oxidizing PMSO in the ZVI/Cu-PMS system. $[PMS]_0$ = 1 mM, $[catalyst]_0$ = 100 mg L^{-1}, $[PMSO]_0$ = 0.1 mM, initial pH = 3 ± 0.2, T = 25±1 ^oC.

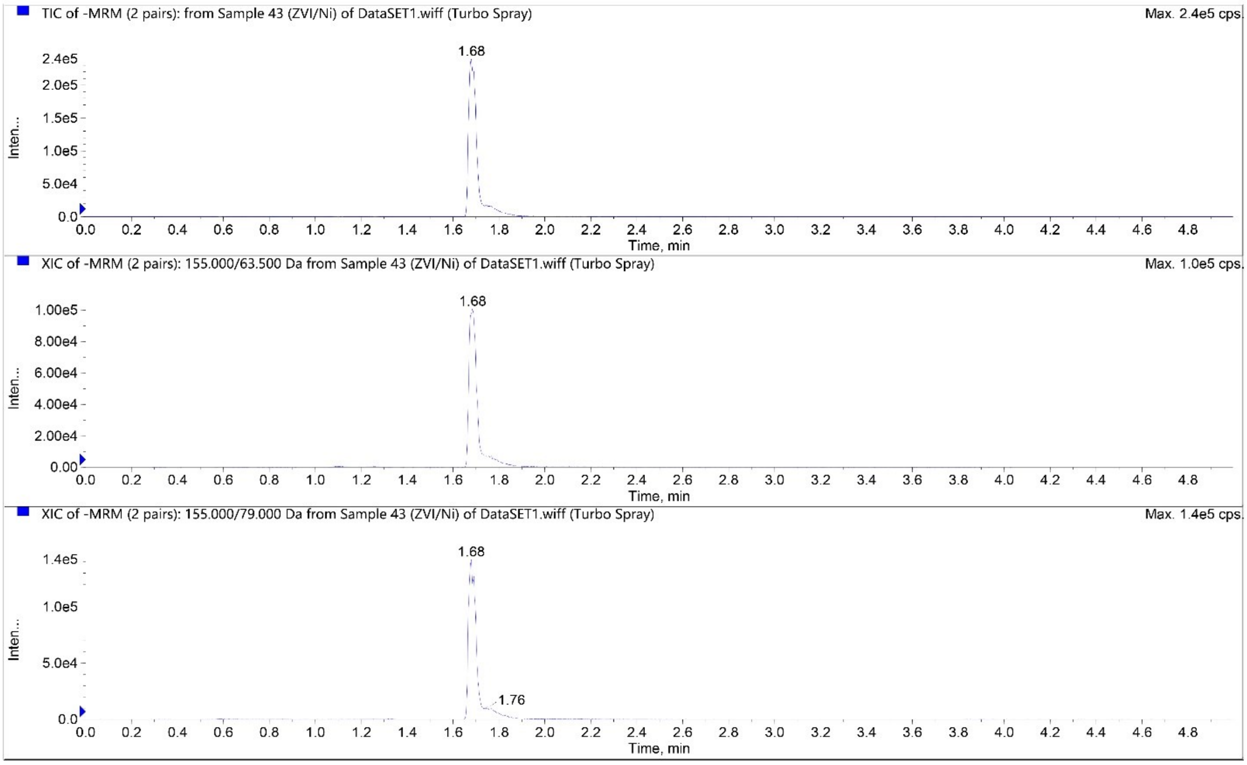

Fig. S11. UPLC/ESI-MS/MS chromatograms of unlabled $PMSO_2$ produced by oxidizing PMSO in the ZVI/Ni-PMS system. $[PMS]_0$ = 1 mM, $[catalyst]_0$ = 100 mg L^{-1}, $[PMSO]_0$ = 0.1 mM, initial pH = 3 ± 0.2, T = 25±1 ^oC.

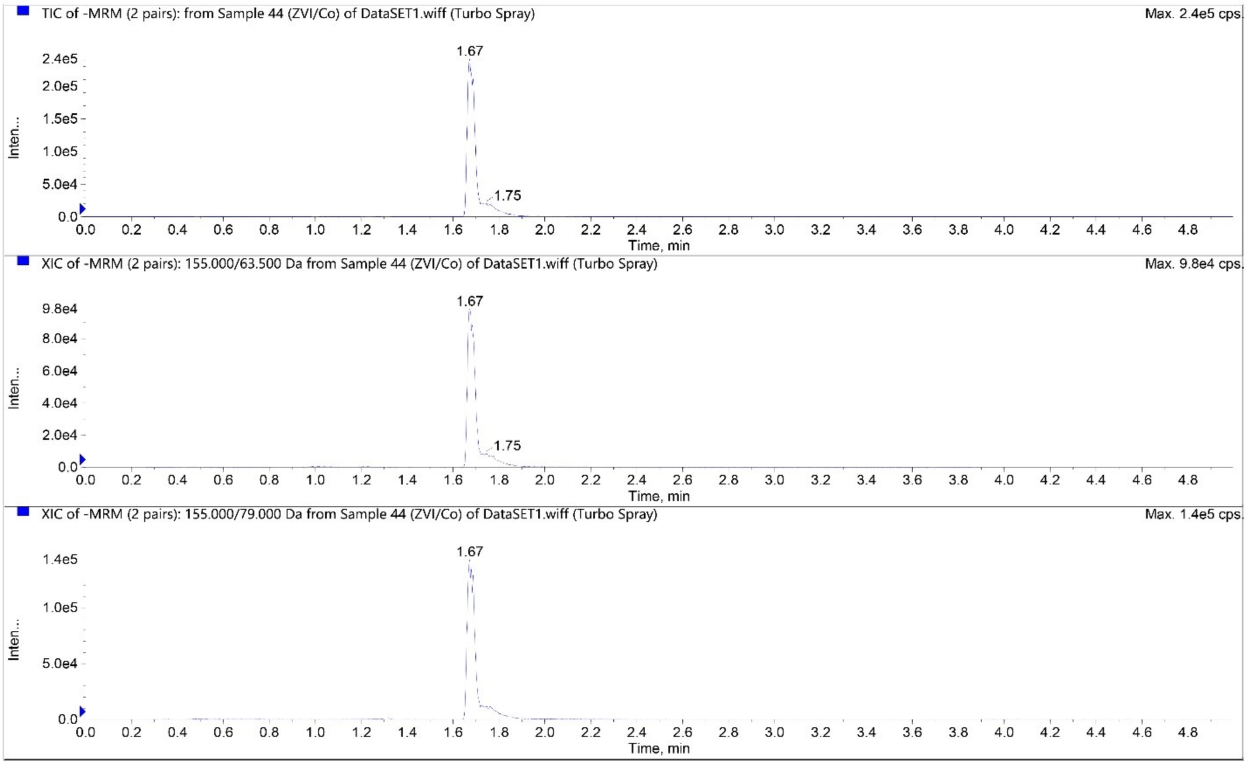

Fig. S12. UPLC/ESI-MS/MS chromatograms of unlabled $PMSO_2$ produced by oxidizing PMSO in the ZVI/Co-PMS system. $[PMS]_0$ = 1 mM, $[catalyst]_0$ = 100 mg L^{-1}, $[PMSO]_0$ = 0.1 mM, initial pH = 3 ± 0.2, T = 25±1 ^oC.

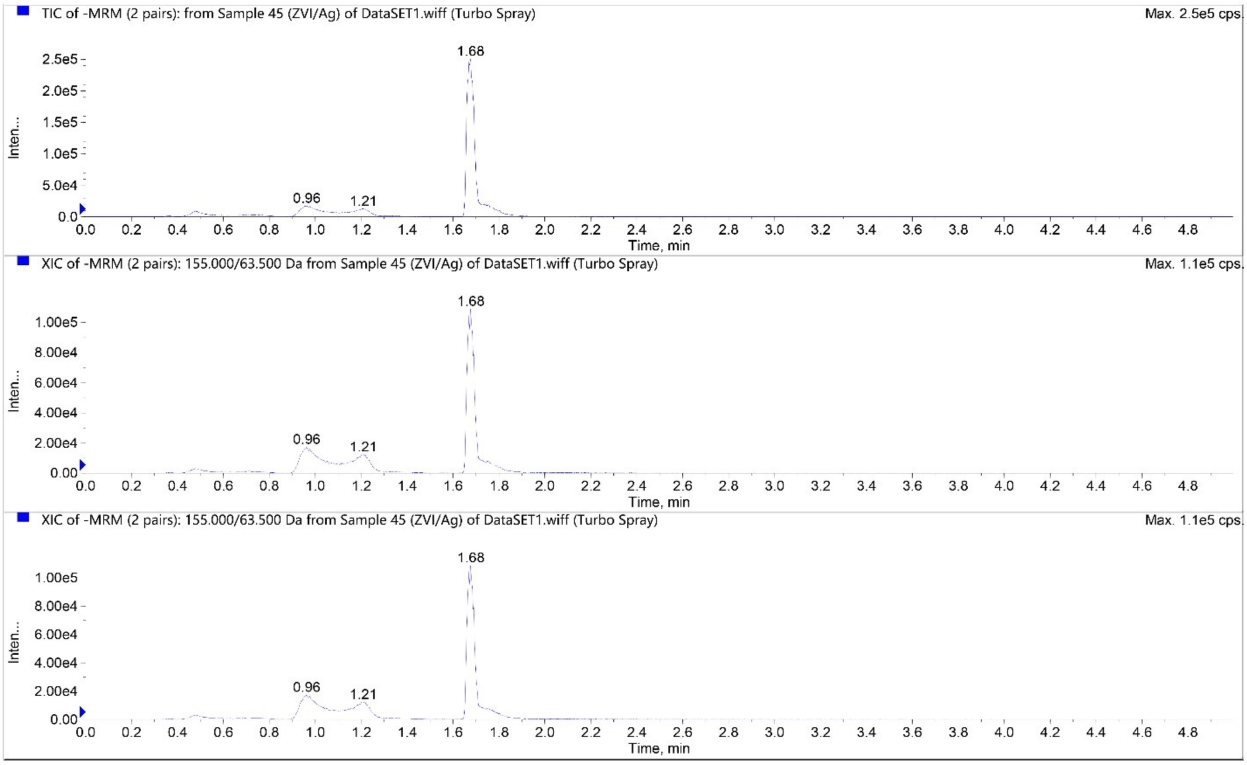

Fig. S13. UPLC/ESI-MS/MS chromatograms of unlabled $PMSO_2$ produced by oxidizing PMSO in the ZVI/Ag-PMS system. $[PMS]_0$ = 1 mM, $[catalyst]_0$ = 100 mg L^{-1}, $[PMSO]_0$ = 0.1 mM, initial pH = 3 ± 0.2, T = 25±1 ^oC.

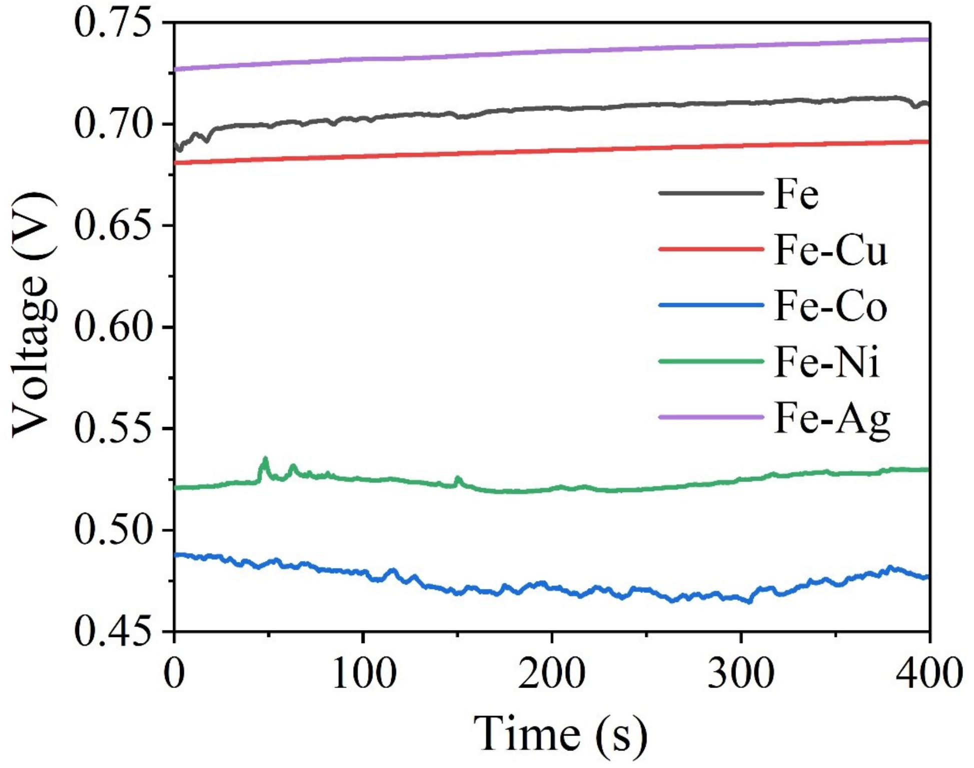

Fig. S14. Open-circuit potential curves on the glassy carbon electrodes in different systems.

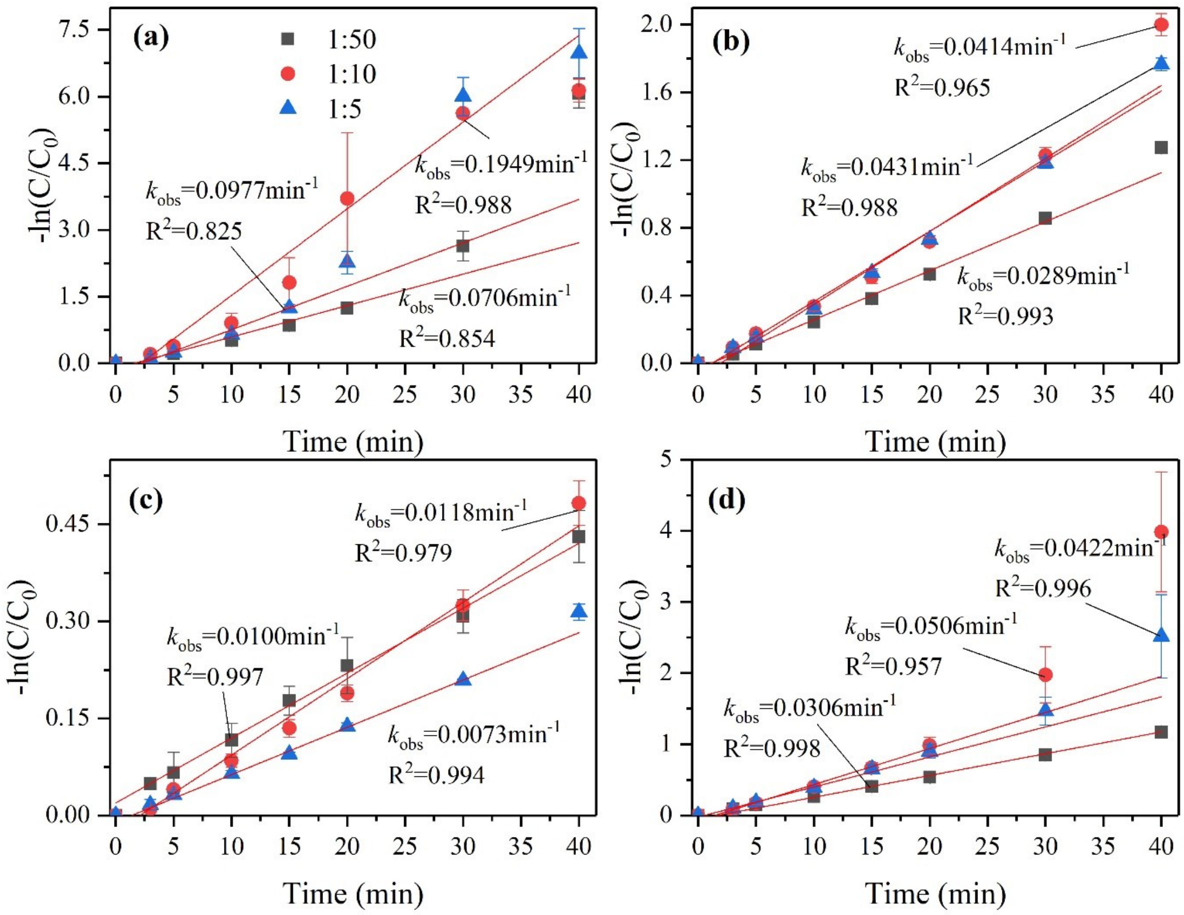

Fig. S15. The reaction rate with different M: Fe ratio using the catalyst as (a) Fe/Cu, (b) Fe/Co, (c) Fe/Ni and (d) Fe/Ag. $[RB]_0$ = 20 mg L^{-1}, $[PMS]_0$ = 1 mM, [PMS]: [RB] =15.4:1, $[catalyst]_0$ = 100 mg L^{-1}, initial pH = 3 ± 0.2, T = 25±1 ^{o}C.

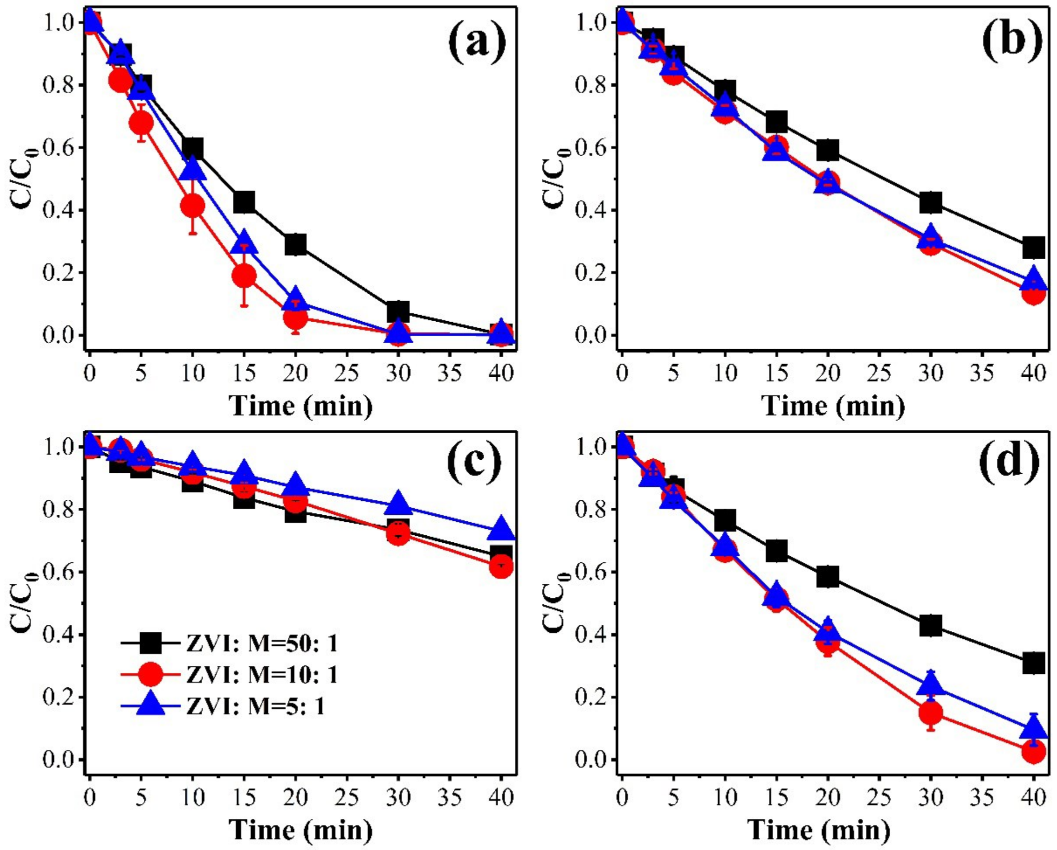

Fig. S16. RB degradation when the bimetallic ratio (M: ZVI) is 1: 50, 1: 10 and 1: 5 using the catalyst as (a) ZVI/Cu, (b) ZVI/Co, (c) ZVI/Ni and (d) ZVI/Ag within 20 min. $[RB]_0$ = 20 mg L^{-1}, $[PMS]_0$ = 1 mM, [PMS]: [RB] =15.4:1, $[catalyst]_0$ = 100 mg L^{-1}, initial pH = 3 ± 0.2, T = 25±1 ^oC.

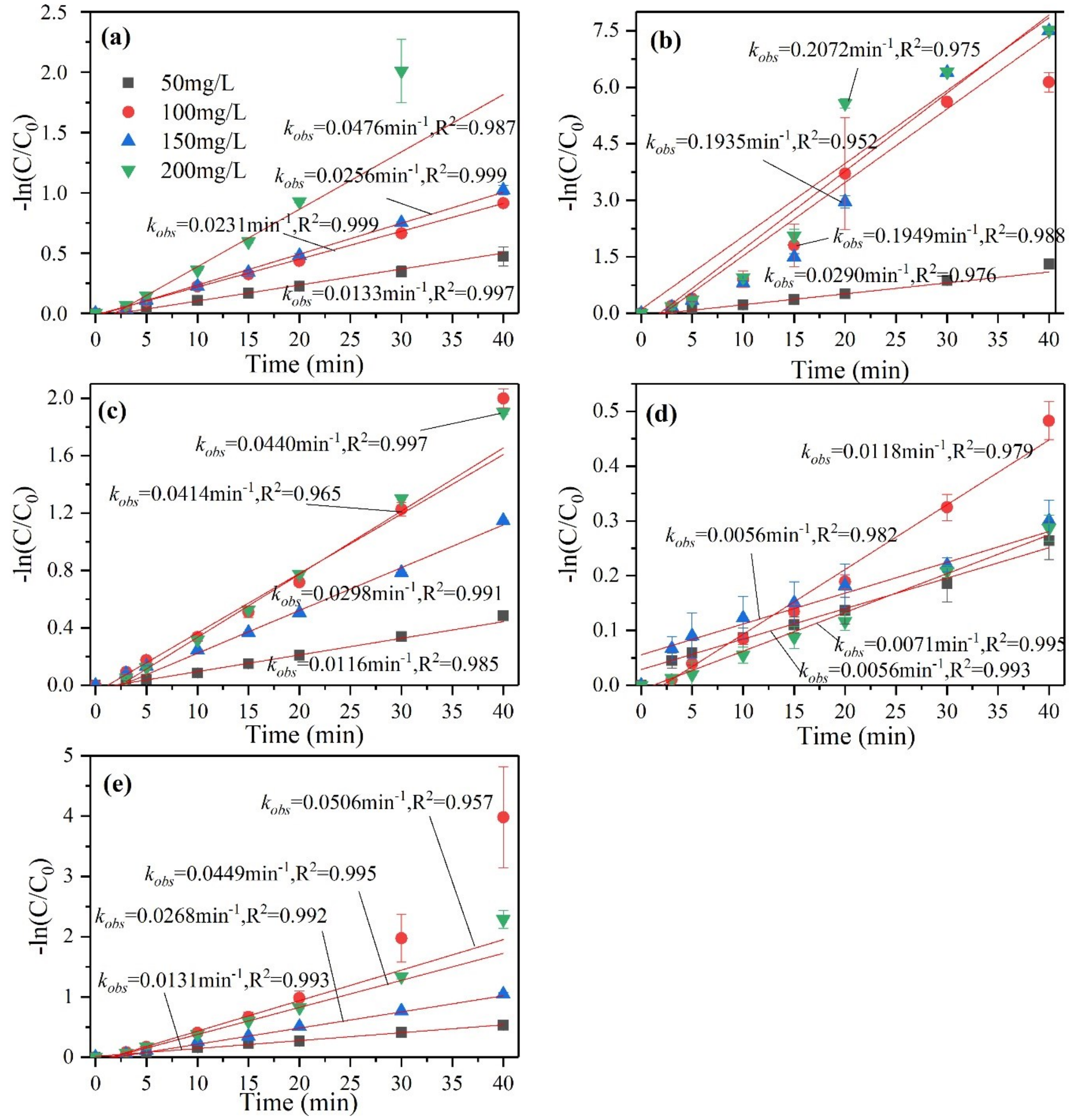

Fig. S17. The reaction rate with different catalysts dosages using the catalysts as (a) Fe, (b) Fe/Cu, (c) Fe/Co, (d) Fe/Ni and (e) Fe/Ag. $[RB]_0$ = 20 mg L^{-1}, $[PMS]_0$ = 1mM, [PMS]: [RB] =15.4:1, initial pH = 3 ± 0.2, T = 25±1 ^oC.

Table. S2. Characteristics of various water matrices.

Water	DW	JAW	LW	MYW	RW
pH	5.77	8.01	7.51	7.79	8.16
TOC	-	1.13±0.10	1.78±0.05	1.80±0.42	0.60±0.04
F^- (mg L^{-1})	-	0.143	0.445	0.204	0.256
Cl^- (mg L^{-1})	-	3.778	8.077	6.936	7.111
NO_2^- (mg L^{-1})	-	5.300	4.075	5.240	4.380
NO_3^- (mg L^{-1})	-	3.246	0.792	0.930	2.521
Na^+ (mg L^{-1})	-	5.004	8.566	7.594	3.308
NH_4^+ (mg L^{-1})	-	1.419	1.082	1.607	1.054
K^+ (mg L^{-1})	-	5.289	2.455	2.229	3.770
Mg^{2+} (mg L^{-1})	-	8.180	11.161	10.963	7.185
Ca^{2+} (mg L^{-1})	-	34.566	21.341	32.976	31.488
Sr^{2+} (mg L^{-1})	-	7.437	6.868	7.440	0.570

Note: “-” stands for undetected.

References

1. Z. Wang, W. Qiu, S.-y. Pang, Y. Zhou, Y. Gao, C. Guan and J. Jiang, *Chemical Engineering Journal*, 2019, **371**, 842-847.
2. B. Yang, P. Zhou, X. Cheng, H. Li, X. Huo and Y. Zhang, *Journal of colloid and interface science*, 2019, **555**, 383-393.
3. L. Hu, G. Zhang, M. Liu, Q. Wang and P. Wang, *Chemical Engineering Journal*, 2018, **338**, 300-310.
